# Exploring agricultural land-use and childhood malaria associations in sub-Saharan Africa

**DOI:** 10.1038/s41598-022-07837-6

**Published:** 2022-03-08

**Authors:** Hiral Anil Shah, Luis Roman Carrasco, Arran Hamlet, Kris A. Murray

**Affiliations:** 1 MRC Centre for Global Infectious Disease Analysis, Department of Infectious Disease Epidemiology, School of Public Health, Imperial College London, London, UK; 2 Grantham Institute – Climate Change and the Environment – Imperial College London, London, UK; 3 Department of Biological Sciences, National University of Singapore, Singapore, Singapore; 4 MRC Unit The Gambia at London, School of Hygiene and Tropical Medicine, Atlantic Boulevard, Fajara, The Gambia; 5 Centre on Climate Change and Planetary Health, London School of Hygiene & Tropical Medicine, London, UK

**Keywords:** Ecological epidemiology, Malaria, Forest ecology, Agroecology, Risk factors, Epidemiology, Sustainability

## Abstract

Agriculture in Africa is rapidly expanding but with this comes potential disbenefits for the environment and human health. Here, we retrospectively assess whether childhood malaria in sub-Saharan Africa varies across differing agricultural land uses after controlling for socio-economic and environmental confounders. Using a multi-model inference hierarchical modelling framework, we found that rainfed cropland was associated with increased malaria in rural (OR 1.10, CI 1.03–1.18) but not urban areas, while irrigated or post flooding cropland was associated with malaria in urban (OR 1.09, CI 1.00–1.18) but not rural areas. In contrast, although malaria was associated with complete forest cover (OR 1.35, CI 1.24–1.47), the presence of natural vegetation in agricultural lands potentially reduces the odds of malaria depending on rural–urban context. In contrast, no associations with malaria were observed for natural vegetation interspersed with cropland (veg-dominant mosaic). Agricultural expansion through rainfed or irrigated cropland may increase childhood malaria in rural or urban contexts in sub-Saharan Africa but retaining some natural vegetation within croplands could help mitigate this risk and provide environmental co-benefits.

## Introduction

Although sub-Saharan Africa has made remarkable gains in many areas of health, such as reduced smoking rates and reductions in maternal mortality, the region has many continuing health challenges to resolve, particularly at the interface of the environment and health^1–3^. Foremost among them is the elimination and eradication of malaria. Although there have been several years of continuous decline in malaria prevalence and incidence across Africa, reductions in malaria cases have recently stalled. Africa accounts for 93% of all malaria cases worldwide, among which children aged under 5 years are the most vulnerable group^4,5^.

At the same time, the current population across sub-Saharan Africa is projected to roughly quadruple to ~ 4 billion by 2100, with much of this growth occurring in rural areas^6–8^. Such growth places considerable demand on the region’s food supply and governments are now considering or implementing large-scale agricultural projects to meet this increased need. At the same time, increasing international trade in agricultural products is a favoured development objective but similarly contributes to further agricultural expansion and its corresponding impacts such as deforestation, which has in turn been linked to malaria risk^9,10^. Hence, agricultural development may undermine efforts to eliminate malaria; for example, by bolstering mosquito populations or accelerating chemical use and resistance^6,11–13^. Although there is considerable urban to rural migration in the region, projections suggest that unescapable circular migration of urbanites to rural areas during crop growing seasons to generate income and reduce dependence on the market for food will continue into the future^8^. Agriculture may therefore continue to influence malaria risk in the future and limit achievement of specific malaria related global goals^6,14^.

Better resolving the links between differing agricultural land-uses and malaria risk in humans could help policy makers identify to what extent expansion of specific agricultural land uses may impact malaria in the region, thereby improving agricultural productivity and sustainability^14^. For instance, expansion of differing agricultural land uses may have varied impacts on habitat suitability of specific malaria transmitting mosquitoes and subsequent malaria transmission^15^. Frontier malaria may also occur, where a change in spatial or temporal malaria risk of previously undeveloped areas occurs as a result of large-scale land-use transformations due to agriculture^15–21^.

Many studies have previously focussed on the relationships between agriculture and malaria vectors or parasites^15,22–26^. Some studies have also focussed on the links between water movement such as dam construction for agriculture and malaria^27–32^. However, fewer studies have assessed the relationships between differing agricultural land uses and malaria outcomes in humans^6,26,33–35^, and those that have tend to be of limited spatial scale with minimal controlling for confounding factors such as socio-economics, demographics (e.g., urban vs rural) or climate. For example, Ijumba et al. found that the incidence of clinical episodes of malaria in Tanzania was significantly less in children living close to a large area of irrigated rice production than in other communities without rice^33^, while Klinkenberg et al. found urban agriculture was marginally associated with childhood malaria risk in Ghana^35^. In contrast, Janko et al. found that increased exposure to agriculture increased malaria risk in children younger than 5 years across rural settings^6^. However, here the authors did not assess the relationship between childhood malaria and specific agricultural land use types (e.g. irrigated vs rainfed croplands or cropland with and without areas of natural vegetation)^6^, limiting the extent to which the results can inform agricultural development and land-use decision making.

Here, we aimed to explore and quantify associations between differing agricultural land uses and childhood malaria across Sub-Saharan Africa. Specifically, we combined remotely-sensed land cover and land use data with a large geo-referenced malaria dataset from the Demographic and Health Surveys (DHS), comprising 24,034 children across 12 countries, to ask: what impact does increasing exposure to differing agricultural land cover types (including rainfed, irrigated/post flooding and cropland-natural vegetation mosaics with varying levels of coverage (i.e., dominated by either cropland, hereafter crop-dominant mosaics, or natural vegetation, hereafter veg-dominated mosaics)) have on the odds of childhood malaria in rural and urban households across sub-Saharan Africa?

We use a multi-model inference hierarchical modelling framework to retrospectively assess relationships between malaria infection status and agricultural covariates while controlling for a number of important individual (age and sex of child), household (education level of the mother, wealth of household, access to improved sanitation and water sources, whether the child slept in a bed-net and whether the dwelling was sprayed with insecticide within the last 12 months)^36^ and extrinsic or environmental confounders (population density, forest cover, forest loss, temperature, precipitation and elevation)^18,37–44^.

## Results

### Descriptive analysis and multicollinearity

**Figure 1:**
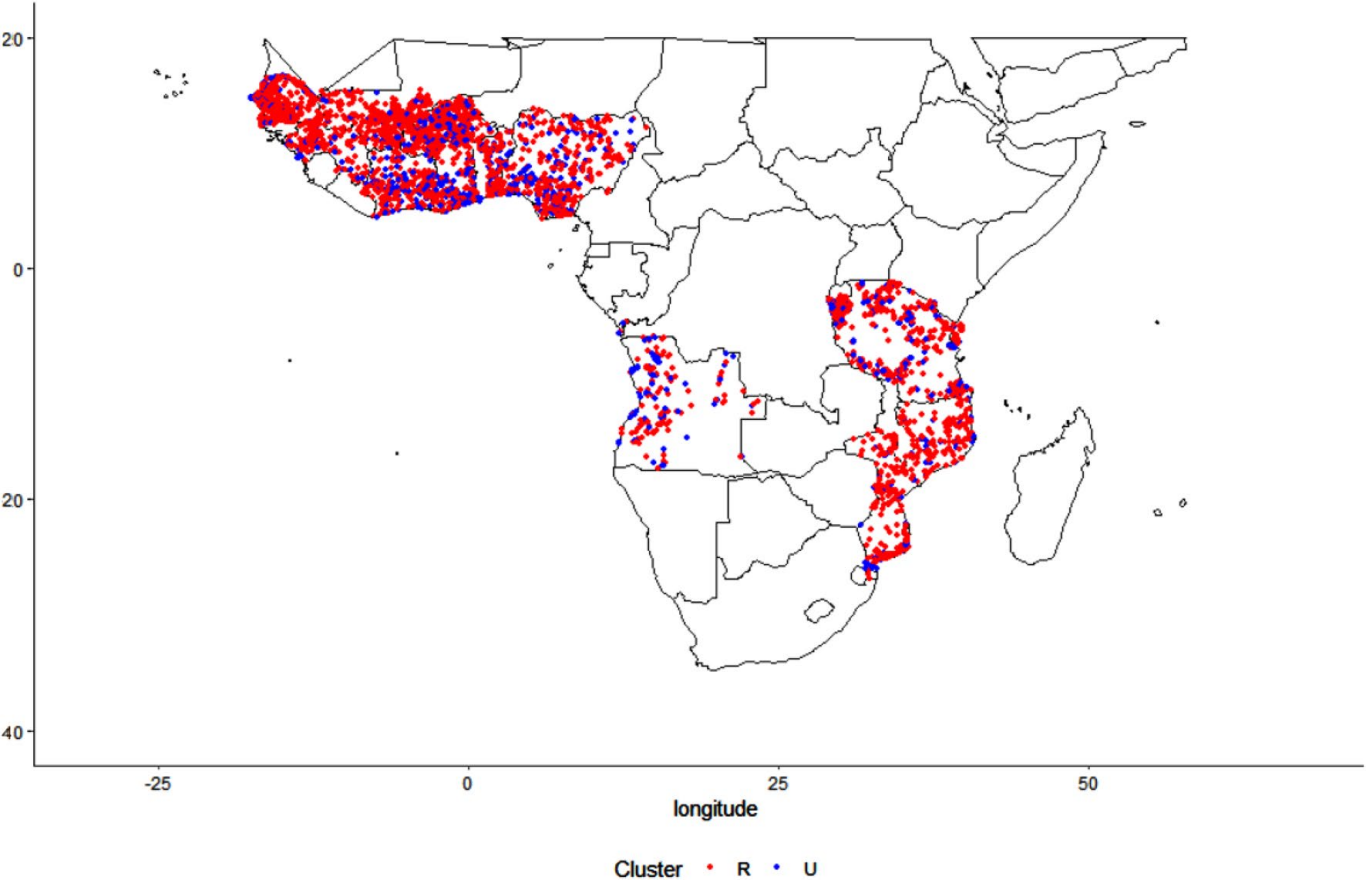
Location of household rural and urban clusters. Our georeferenced dataset includes 24,034 children in 14,281 households in 4028 clusters located in 12 countries between 2010 and 2015. The dataset links geo-referenced Demographic and Health Surveys (DHS) individual and household information with data on agricultural land uses, forest cover, forest loss and climate.

Our final data set sourced from the DHS consisted of 24,034 individuals in 14,281 households in 4028 clusters located in 12 countries (Fig. 1). Of these individuals, 22.14% tested positive for malaria using either a blood smear test (BST) or rapid diagnostic test (RDT) (Table 1). Data were collected between the years of 2010 until 2015, with 12.42% of samples collected in 2010, 11.53% in 2011, 24.89% in 2012, 7.34% in 2013, 9.41% in 2014 and 34.42% in 2015.

**Table 1:** Descriptive statistics.

	Malaria (-ve)	Malaria (+ ve)
**Socioeconomic variables**		
Total sample (n) (%)	18,712 (77.85%)	5322 (22.14%)
Age (years) (VIF = 1.03)		
Mean	2.35	2.52
SD	1.49	1.39
Cluster type (n) (%) (VIF = 1.39)		
Urban	7500 (40.08%)	1126 (21.16%)
Rural	11,212 (59.92%)	4196 (78.84%)
Country (n) (%)		
Angola	1176 (6.28%)	184 (3.46%)
Burkina Faso	417 (2.23%)	843 (15.84%)
Benin	958 (5.12%)	260 (4.89%)
Burundi	1800 (9.62%)	227 (4.27%)
Cote D’Ivoire	637 (3.40%)	407 (7.65%)
Ghana	344 (1.84%)	273 (5.13%)
Guinea	391 (2.09%)	167 (3.14%)
Mali	900 (4.81%)	466 (8.76%)
Mozambique	1261 (6.74%)	634 (11.91%)
Nigeria	1901 (10.16%)	1448 (27.21%)
Senegal	5906 (31.56%)	129 (2.42%)
Tanzania	3021 (16.14%)	284 (5.34%)
Dwelling sprayed in last 12 months (n) (%) (VIF = 1.02)		
Yes	16,980 (90.74%)	5034 (94.59%)
No	1732 (9.26%)	288 (5.41%)
Mothers education (n) (%) (VIF = 1.02)		
No education	18,514 (98.94%)	5283 (99.27%)
Primary	196 (1.05%)	38 (0.71%)
Secondary and Higher	2 (0.01%)	10.02%)
Population density ((number of persons per km^2^) (VIF = 1.34)		
Mean	858.94	303.67
SD	2106.17	867.49
Sanitation (n) (%) (VIF = 1.79)		
Improved	11,355 (60.68%)	2417 (45.42%)
Unimproved	7357 (39.31%)	2905 (54.58%)
Sex (n) (%) (VIF = 1.00)		
Female	9461 (50.56%)	2743 (51.54%)
Male	9251 (49.43%)	2579 (48.46%)
Used a bed net (n) (%) (VIF = 1.02)		
Did not use a bed net	7501 (40.09%)	2273 (42.71%)
Some children used a bed net	2152 (11.50%)	663 (12.46%)
All children used a bed net	9059 (48.41%)	2386 (44.83%)
Wealth index (n) (%) (VIF = 1.42)		
1 = Poorest	3390 (18.12%)	1386 (26.04%)
2 = Poorer	3519 (18.81%)	1309 (24.60%)
3 = Middle	3739 (19.98%)	1244 (23.37%)
4 = Richer	4097 (21.90%)	903 (16.97%)
5 = Richest	3967 (21.20%)	480 (9.02%)
Water source (n) (%) (VIF = 1.92)		
Improved	9359 (50.02%)	1063 (19.97%)
Unimproved	9353 (49.98%)	4259 (80.03%)
Year (n) (%) (VIF = 1.09)		
2010	1578 (8.43%)	1407 (26.44%)
2011	2087 (11.15%)	683 (12.83%)
2012	4641 (24.80%)	1340 (25.18%)
2013	1549 (8.28%)	215 (4.04%)
2014	1966 (10.51%)	296 (5.56%)
2015	6891 (36.83%)	1381 (25.95%)
**Environmental variables**		
Elevation (m) (VIF = 1.58)		
Mean	453.54	392.50
SD	595.10	367.20
Forest loss (%) (VIF = 1.16)		
Mean	0.15	0.18
SD	0.36	0.43
Mean temperature (°C) (VIF = 1.48)		
Mean	24.93	25.33
SD	3.47	2.59
Precipitation (mm) (VIF = 1.13)		
Mean	77.57	86.09
SD	91.77	98.48
**Agricultural land use variables**		
Crop-dominated mosaics (%) (VIF = 1.16)		
Mean	5.96	7.31
SD	9.22	9.19
Forest cover (%) (VIF = 1.38)		
Mean	12.84	16.41
SD	13.42	16.25
Irrigated/post-flooding cropland (%) (VIF = 1.10)		
Mean	2.53	2.77
SD	9.37	11.46
Rainfed cropland (%) (VIF = 1.15)		
Mean	26.29	35.17
SD	27.84	30.53
Veg-dominated mosaics (%) (VIF = 1.22)		
Mean	3.57	5.93
SD	6.52	9.49

Descriptive statistics of all variables included in the geo-referenced dataset. VIF denotes Variance Inflation Factor.

Countries include Angola (5.66%), Burkina Faso (5.24%), Benin (5.07%), Burundi (8.43%), Cote D’Ivoire (4.34%), Ghana (2.57%), Guinea (2.32%), Mali (5.68%), Mozambique (7.88%), Nigeria (13.93%), Senegal (25.11%) and Tanzania (13.75%). No variables within our dataset presented problems of multicollinearity. We therefore included all variables for analysis (Table 1). Further details including a data flow diagram, additional descriptive results and a correlation matrix of all variables can be found in Supplementary Information Dataset 1.

### Sub-Saharan Africa multivariate analysis

**Figure 2:**
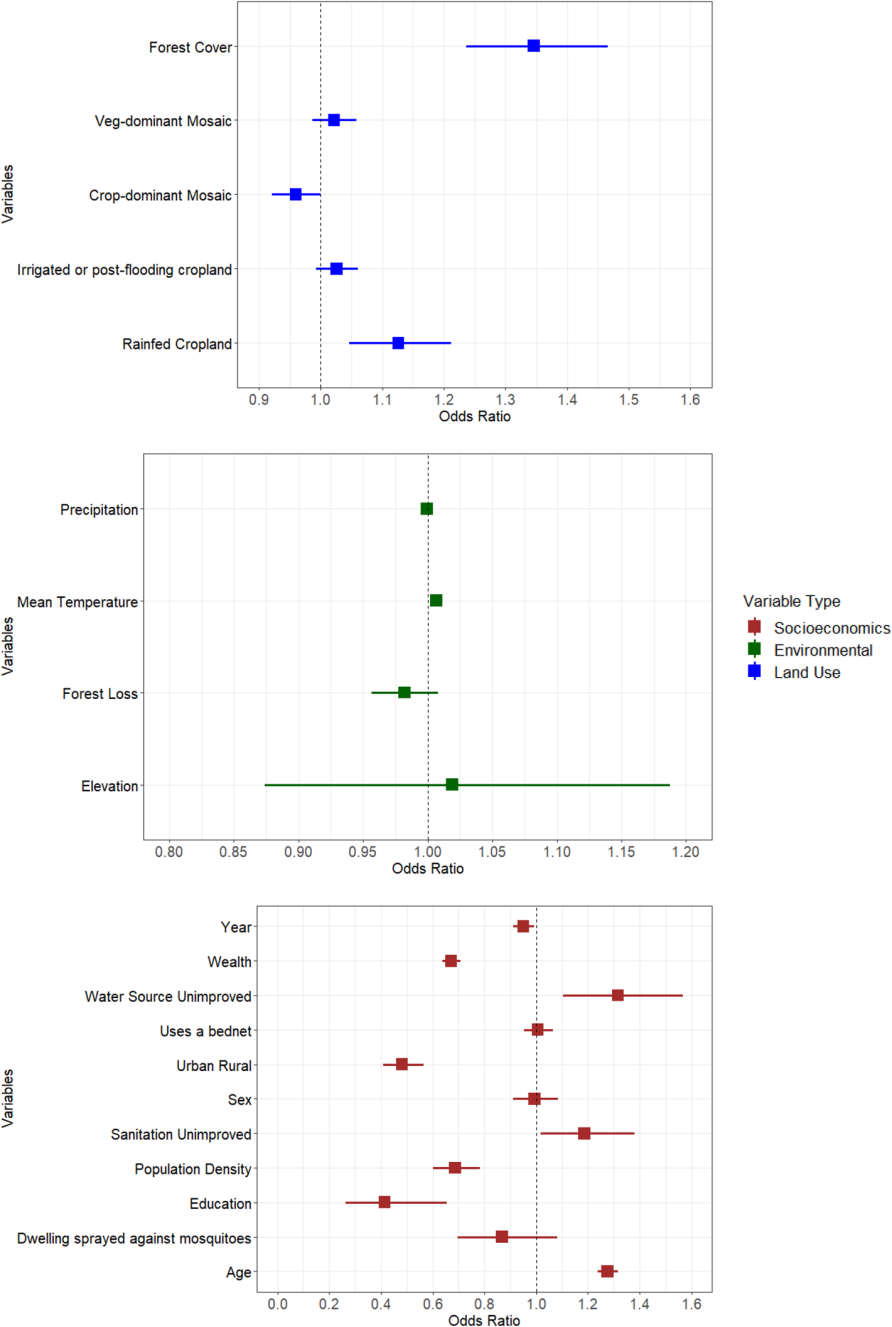
Sub-Saharan regional multivariate analysis—a multivariate analysis that assesses the factors associated with the odds of childhood malaria. Error bars are defined as the 95% confidence interval. Variables increasing childhood malaria have odds ratios greater than 1 to the right of the vertical line. Crop-dominated mosaic denotes mosaic cropland and veg-dominated mosaic denotes mosaic natural vegetation within the European Space Agency (ESA) Climate Change Initiative Land Cover (CCI-LC) dataset.

At the regional level across all 12 countries, a “U-Shaped” relationship was found across land use classes and the odds of childhood malaria, when ordered to reflect the transitions from natural to fully converted land; that is, natural forest cover (highest odds) to mosaics (lowest odds) through to intensive agriculture (intermediate odds) (Fig. 2). Here, the greatest odds were observed for exposure to complete forest cover (no agricultural land use), which was associated with 35% (OR 1.35, CI 1.24–1.47) increased odds of childhood malaria. In contrast, mosaic vegetation structures that include cropland but remain dominated by natural vegetation were not associated with childhood malaria, and mosaics that are dominated by crops but also include natural vegetation were marginally negatively associated with the odds of childhood malaria (OR 0.96, 0.92–1.00). This represents the only land use class that potentially neutralises or even reduces the odds of childhood malaria overall. Exposure to irrigated or post-flooding cropland resulted in a generally positive but non-significant association with the odds of childhood malaria (OR 1.03, CI 0.99–1.06), while exposure to rainfed cropland was significantly associated with increased childhood malaria (OR 1.13, CI 1.05–1.21).

When considering environmental confounders, no significant associations were found between malaria infection and forest loss (OR 0.98, CI 0.96–1.01), mean temperature (OR 1.01, CI 1.00–1.01), precipitation (OR 1.00, CI 1.00–1.00), or elevation (OR 1.02, CI 0.87–1.19) (Fig. 2). With respect to potential socio-economic and other confounders, unimproved water sources and unimproved sanitation were associated with a 32% (OR 1.32, CI 1.11–1.57) and 19% (OR 1.19, CI 1.02–1.38) increased odds of childhood malaria when compared to improved water sources or sanitation, respectively. Age (OR 1.28, CI 1.24–1.32) was also positively associated with a higher odds of childhood malaria. Higher maternal education (OR 0.41, CI 0.26–0.65), wealth (OR 0.67, CI 0.64–0.71) and population density (OR 0.69, CI 0.60–0.78) were all associated with considerable reductions in malaria infection. Urban areas had a considerable reduction in the odds of childhood malaria compared to rural areas (OR 0.48, CI 0.41–0.57) (Fig. 2). Finally, no associations were found between the odds of childhood malaria and spraying the dwelling with insecticide (OR 0.87, CI 0.70–1.08), using a bed net (OR 1.00, CI 0.95–1.06) or a child’s sex (OR 0.99, CI 0.91–1.09) (Fig. 2). Full details of these results are summarised in Supplementary Information Table S1.

### Univariate sensitivity analysis

**Figure 3:**
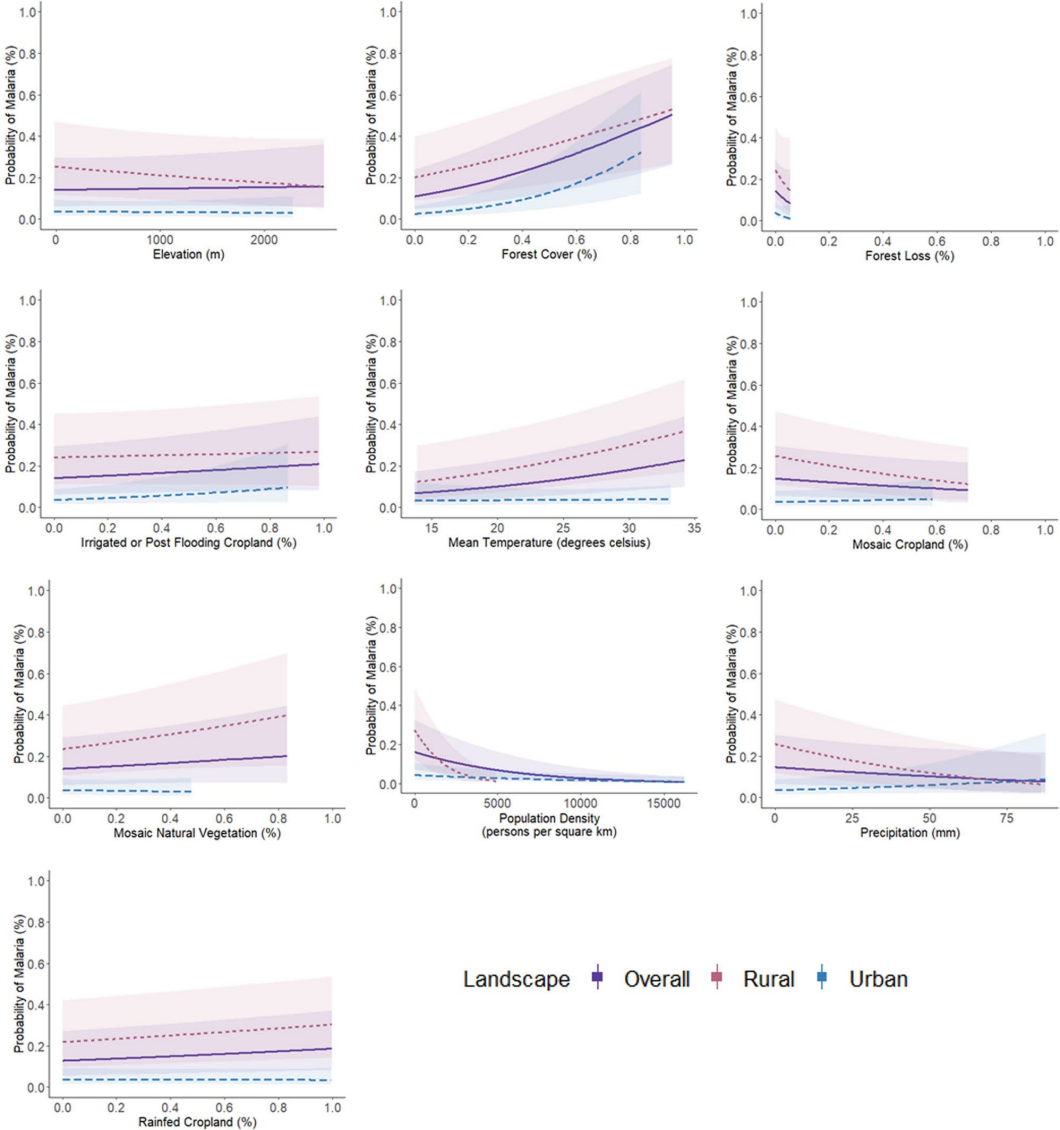
Univariate sensitivity analysis for continuous predictors. Marginal effects curves for continuous predictors included within the global model. This is a univariate sensitivity analysis that generates predictions generated by a model when one holds the non-focal variables constant and varies the focal variable. The global model consists of all variables within our georeferenced dataset and represents the most complex model. Marginal effects measure the instantaneous effect that a change in a particular explanatory variable has on the predicted probability of malaria when the other covariates are kept fixed.

The marginal effect of a continuous independent variable (e.g. rainfed cropland) is the instantaneous rate of change (e.g. the change in the probability of malaria) given very small changes (close to zero) in that variable^45^. Here, results suggest that the global model was most sensitive to forest cover and population density with the instantaneous rate of change in the probability of malaria increasing and decreasing substantially for these two predictors, respectively (Fig. 3) (note, here forest cover does not reflect reforestation but rather the static extent of forest cover in the dataset).

**Figure 4:**
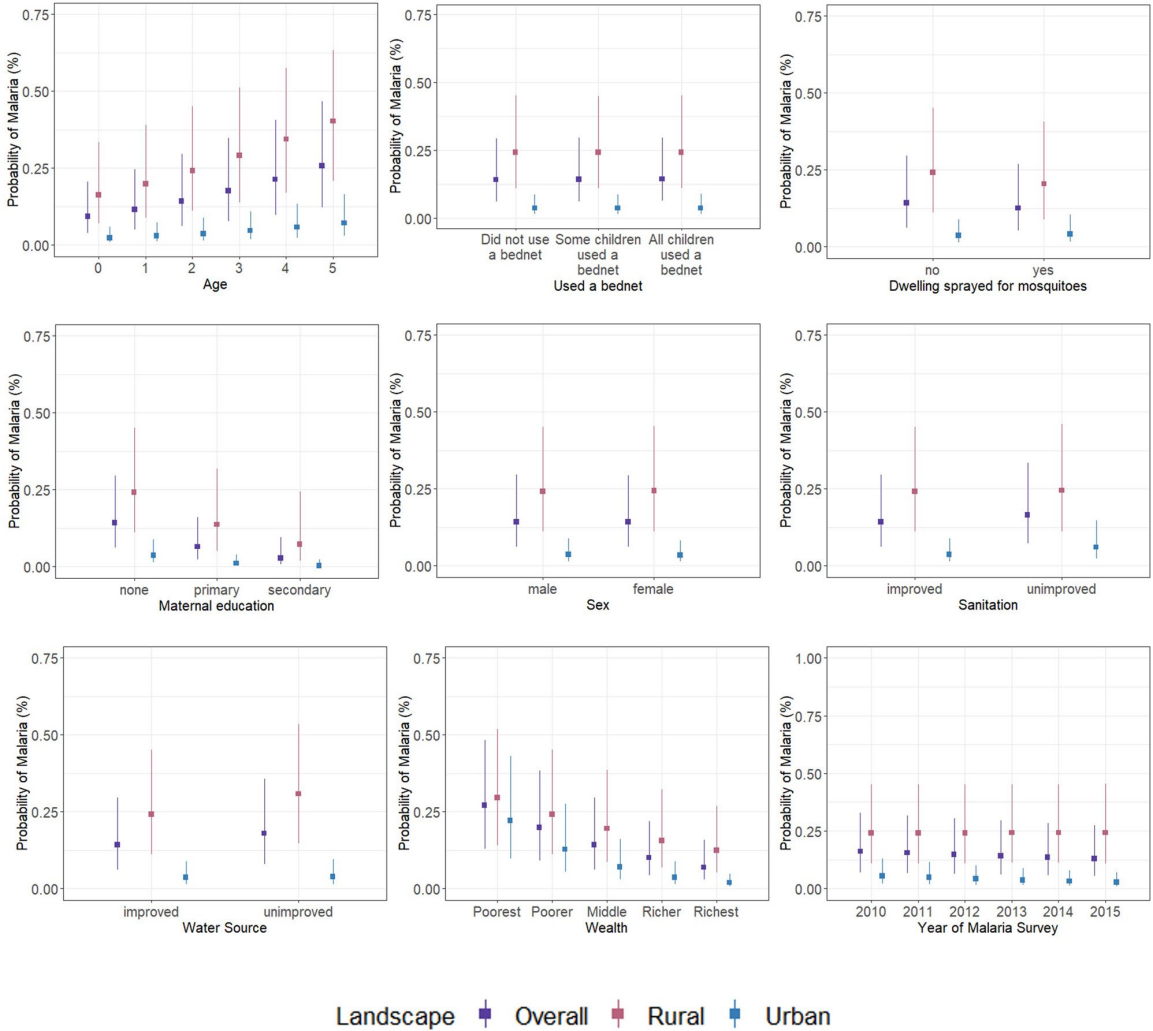
Univariate sensitivity analysis for discrete predictors. Marginal effects curves for discrete predictors included within the global model. This is a univariate sensitivity analysis that generates predictions generated by a model when one holds the non-focal variables constant and varies the focal variable. The global model consists of all variables within our georeferenced dataset and represents the most complex model. Marginal effects measure the instantaneous effect that a change in a particular explanatory variable has on the predicted probability of malaria when the other covariates are kept fixed.

For the human-modified landscape variables, the probability of malaria steadily increased when small increases were made in rainfed, irrigated or post-flooding cropland and veg-dominated mosaics (Fig. 3). When stratifying by cluster type, we found that childhood malaria risk was most sensitive to continuous predictors especially in rural clusters thereby warranting subgroup analysis. Marginal effects for socio-economic interventions were consistent and expected at the overall level (Fig. 4).

### Stratified multivariate analysis of urban and rural households

**Figure 5:**
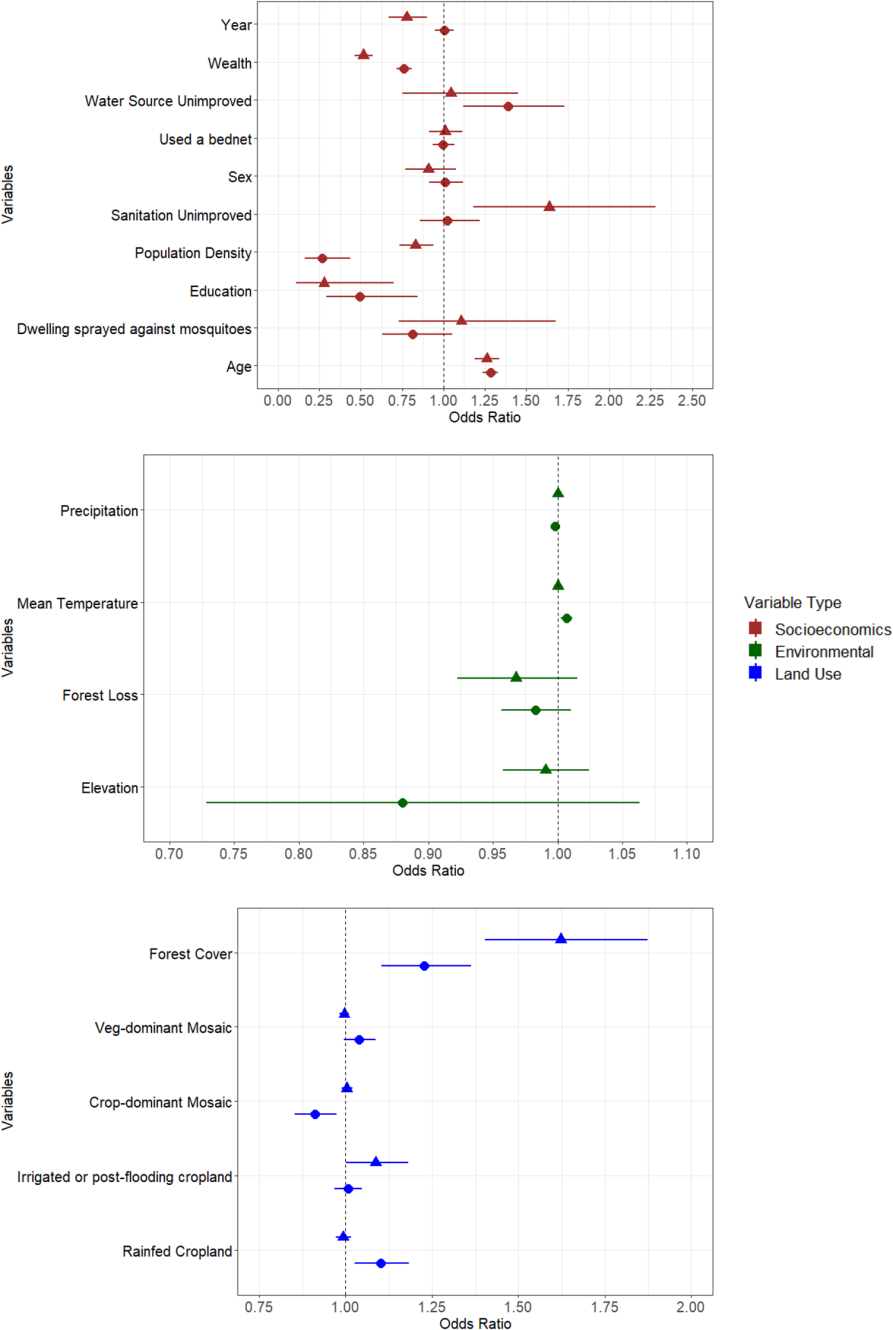
Stratified multivariate analysis of rural and urban households. Factors associated with the odds of childhood malaria differ between rural and urban households. Error bars are defined as the 95% confidence interval. Variables increasing childhood malaria have odds ratios greater than 1 to the right of the vertical line. Crop-dominated mosaic denotes mosaic cropland and veg-dominated mosaic denotes mosaic natural vegetation within the European Space Agency (ESA) Climate Change Initiative Land Cover (CCI-LC) dataset.

The overall effects of differing agricultural land uses did in some cases vary considerably between rural and urban clusters, as shown by the subgroup analysis (Fig. 5). Specifically, a positive association was found for rainfed cropland in rural clusters (OR 1.10, CI 1.03–1.18) but not in urban areas (OR 0.99, CI 0.97–1.01). On the other hand, irrigation or post-flooding cropland was marginally associated with the odds of childhood malaria in urban clusters (OR 1.09, CI 1.00–1.18) but not in rural areas (OR 1.01, CI 0.97–1.05). In rural clusters, crop-dominant mosaics (OR 0.91, CI 0.85–0.97) were negatively associated with the odds of childhood malaria, whereas veg-dominant mosaics in rural clusters were not associated with childhood malaria (OR 1.04, CI 0.99–1.09). No effect was found for either mosaic classes in urban clusters (veg-dominant (OR 1.00, CI 0.98–1.01), crop-dominant (OR 1.00, CI 0.99–1.02)).

The overall effect of forest cover was emphasised in urban vs rural clusters (urban: OR 1.62, CI 1.40–1.87; rural: OR 1.23, CI 1.10–1.36) (Fig. 5); however, no association was found between exposure to forest loss and the odds of childhood malaria across urban (OR 0.97, CI 0.92–1.02) or rural clusters (OR 0.98, CI 0.96–1.01). When assessing climatic factors, no associations were found for mean temperature (rural (OR 1.01, CI 1.00–1.01), urban (OR 1.00, CI 0.99–1.00)), precipitation (rural (OR 1.00, CI 1.00–1.00), urban (OR 1.00, CI 1.00–1.00)), or elevation (rural (OR 1.01, CI 1.00–1.01), urban (OR 1.00, CI 0.99–1.00)) across rural or urban clusters.

Associations between childhood malaria and water, sanitation, and hygiene (WaSH) related socio-economic variables also varied across rural and urban households (Fig. 5). Unimproved sanitation in urban households was associated with higher odds of childhood malaria (OR 1.64, CI 1.18–2.28) compared to improved sanitation, whilst no association was found for unimproved sanitation in rural areas (OR 1.02, CI 0.85–1.22). On the other hand, unimproved water sources in rural areas had a higher odds of childhood malaria compared to improved water sources (OR 1.39, CI 1.12–1.73), whereas no association was found in urban areas (OR 1.04, CI 0.75–1.45).

In contrast, consistent patterns were found across both urban and rural settings for maternal education (urban OR 0.28, CI 0.11–0.70; rural OR 0.50, CI 0.29–0.84), wealth (urban OR 0.52, CI 0.46–0.57; rural OR 0.76, CI 0.72–0.81) and population density (urban OR 0.83, CI 0.73–0.94; rural OR 0.27, CI 0.16–0.44), all of which resulted in reduced odds of childhood malaria in both rural and urban clusters (Fig. 5). Age was positively associated with childhood malaria in both rural (OR 1.28, CI 1.24–1.33) and urban clusters (OR 1.26, CI 1.19–1.34). On the other hand, we found that between 2010 and 2015 (equivalent to year of survey), childhood malaria decreased for urban clusters (OR 0.78, CI 0.67–0.90) but not in rural clusters (OR 1.00, CI 0.95–1.06) (Fig. 5). Spraying the dwelling with insecticide (rural (OR 0.81, CI 0.63–1.06), urban (OR 1.11, CI 0.73–1.68)), use of a bed net (rural (OR 1.00, CI 0.93–1.06), urban (OR 1.01, CI 0.92–1.11)) and a child’s sex (rural (OR 1.01, CI 0.91–1.12), urban (OR 0.91, CI 0.77–1.08)) were not associated with the odds of childhood malaria across either rural or urban clusters (Fig. 5). Full results are presented in tabular format in Supplementary Information Table S2.

## Discussion

### Overview

We investigated the impact of land cover on the odds of childhood malaria, a key issue in sub-Saharan Africa at the nexus of agricultural development and human health. We found that childhood malaria was generally associated with exposure to complete forest cover (~ 35%). However, the retention of natural vegetation in cropland (crop-dominated mosaics) potentially reduces or even protects against malaria, especially in rural areas (~ − 9%). On the other hand, rainfed cropland was associated with increased malaria in rural (~ 10%) but not urban areas, while irrigated or post flooding cropland was associated with malaria in urban (~ 9%) but not rural areas. In contrast, no associations with malaria were observed for natural vegetation interspersed with cropland (veg-dominant mosaics). Together, the results suggest a potential role for shifting from rainfed to irrigated cropland in rural areas or for greater natural vegetation in croplands to potentially moderate or even reduce the odds of malaria.

### Agricultural land use and its impact on childhood malaria

Rainfed cropland is the most common agricultural method used by marginal or smallholder subsistence farmers across sub-Saharan Africa. With the sub-Saharan African population projected to quadruple by 2100, there will likely be a vast expansion or intensification of rainfed cropland across the region^46^. Here we find these areas are associated with the highest odds of malaria generally outside forested areas, as well as primarily within rural clusters after controlling for other factors. Mechanisms by which rainfed cropland could influence malaria transmission may include slash and burn practices for shifting agriculture, where an area of ground is cleared of vegetation and cultivated for a few years and then abandoned for a new area until its fertility has been naturally restored^47^. This process could influence malaria transmission through changing habitat suitability of mosquito vectors through increasing sunlight, standing water and high temperatures, which favour some types of malaria transmitting mosquitoes. In addition, rainfed landscapes may also have fewer insectivores, greater competition among remaining species for resources and fewer dead-end hosts to dilute malaria, thereby suggesting that rainfed cropland is particularly impactful on biodiversity^42,48,49^.

Irrigated cropland, on the other hand, only accounts for approximately 6% of all agriculture across sub-Saharan Africa^8^, and therefore represents a relatively limited fraction of our dataset. Nevertheless, we found a marginal positive association between irrigation or post-flooding cropland and childhood malaria within urban clusters but not in rural clusters or at the regional level. Here, previous research suggests that mosquito biting rates increase in urban areas where irrigated farming takes place due to mosquito vectors adapting to more polluted larval habitats, susceptible humans being in near proximity to peri-urban forest cover or the intensive use of pesticides in urban agriculture likely increasing the speed with which insecticide resistance develops^26,35,50–53^. On the other hand, Ijumba and Lindsay (2001) suggest that crop irrigation has little impact on malaria transmission and is dependent on endemic or non-endemic setting status^12^. Such heterogeneity in effects is likely attributable to a range of environmental or socioeconomic factors that may vary geographically (e.g. proximity to peri-urban forests, crop type, humidity, highlands, desert fringes, improvements in wealth, housing, access to care, water, sanitation, seasonality the widespread use of bed-nets and the antimalarials in villages and so on)^1,12,54,55^. Although we provide further evidence on the association between irrigation and childhood malaria in urban areas whilst attempting to account for most of these confounders in our analysis, further research is required to untangle the specific contexts and causal mechanisms linking irrigation to malaria.

Mosaic landscapes, which consist of varying mixes and degrees of cropland or natural vegetation, may occur due to frontier clearing for agricultural, subsistence or smallholder farming, or associated with restoration efforts in previously degraded agricultural areas (e.g., agroforestry). Our results suggest a fine balance in mechanisms that may either increase or decrease disease risk in complex landscapes, depending on majority land-cover classes and their associated factors as well as a potential interaction with rural vs urban status. Specifically, crop-dominated mosaics interspersed with natural vegetation reduced the odds of childhood malaria in rural but not urban areas, while no association was found between veg-dominated mosaic systems and the odds of childhood malaria in either rural or urban clusters. In all mosaic areas, the odds of malaria was lower than for both forested and more intensive cropping areas.

Identifying potential explanations for this intriguing result is a challenge given current knowledge on disease ecology in forested vs artificial vs more complex, potentially fragmented but otherwise more biodiverse landscapes (i.e., compared to crop monocultures). Forest conservation has often been suggested as a potential anti-malarial intervention^42^. However, our analysis suggests that childhood malaria is positively associated with forest cover (natural vegetation) in both rural and urban clusters, yet there is no association between forest loss and childhood malaria^42^. Our results also suggest that childhood malaria is most sensitive to forest cover and population density. The majority of previous research that has explored the links between forest loss, forest cover and malaria has led to contradictory answers with some studies also suggesting that risk or incidence of malaria first increases and then decreases as deforestation proceeds^20,56,57^. Forest disturbance can increase the risk of malaria by increasing human exposure, changing habitat suitability, reducing potential sinks of malaria transmission, or modifying diversity of mosquito species. However, forest loss can also lead to economic development, improved housing, livelihoods, sanitation, education and wealth^48,56,58^.

Given the strong association between forest cover and the odds of childhood malaria, it is perhaps unsurprising to observe lower odds in mosaic landscapes and even a marginal negative association in crop dominated mosaics. However, that this effect increases again in more intensively cropped areas suggests something of a ‘sweet spot’ in terms of overall odds of infection that makes picking apart specific mechanisms of malaria transmission in fragmented landscapes a particular challenge. Previous research suggests that species diversity within mosaic systems can act differently on competing drivers of disease transmission (host density, vector biting rates, vector habitat suitability and transmissibility) and may cause simultaneous increases (amplification) and decreases (dilution) in malaria transmission^59,60^. For example, increasing vegetation can lead to increases in humidity which favours mosquito survival and increases biting rates^61^. Specific crops may be linked to increased (e.g. sweet potatoes or yams) or decreased (e.g. millet) malaria transmission due to respective water or feeding requirements thereby impacting mosquito habitat suitability^62^. Subsistence or small-holder farming can also include the use of livestock, which is known to be a zoo-prophylaxis for malaria^63^. More broadly, dilution effects may be more pronounced in or even restricted to systems in which biodiversity loss generates biodiversity gradients, as opposed to biodiversity gradients generated via other mechanisms^64^.

In any case, until further studies shed greater light on the causes of apparent reductions in malaria in mosaic landscapes as reported here, it remains prudent to prioritize proven anti-malarial interventions such as improvements in education and wealth, insecticide treated nets, spraying of dwellings, and housing improvements. Forest conservation efforts in Africa should instead focus on securing known and proven benefits such as carbon storage, clean water provision, biodiversity, food provision, and other aspects of human health (e.g. diarrheal disease)^42,45,65^, while associated costs including increases in other disease risks, such as malaria, may require more targeted research to resolve and interventions to offset. Nevertheless, targeting less major factors either individually or in combination (e.g., via complex interventions) could represent novel areas to further reduce malaria risk in children in Sub-Saharan Africa. Even relatively small reductions in risk would potentially translate into a large number of lives protected given the ongoing high burden of this disease in this region. This could be important particularly where residual transmission persists despite already strong management intervention efforts. Given the heterogeneity we observed within mosaic systems, to contribute to management further research is required to assess whether such effects are real or spurious and further disentangle how ecological drivers of malaria transmission mechanistically relate to changes in disease risk across landscape types in agricultural systems.

### Environmental and socio-economic confounding

Agricultural-malaria relationships are influenced by many differing environmental and socio-economic factors^66^. Our results are broadly highly consistent with expected confounding effects at the general level and across rural and urban clusters when aiming to isolate the specific effect of agricultural land use, which increases our confidence in the more rarely addressed agricultural land-cover associations reported here. For example, we found no association between environmental confounders such as mean temperature, precipitation or elevation at the general level or across a rural urban stratification^40,42,67–69^. Socioeconomic confounding effects were also generally consistent with previous research, showing that improvements in maternal education^70–72^, wealth^73,74^, sanitation, and water sources^75,76^ alongside increasing urbanisation or population density^77^ all were negatively associated with childhood malaria. Increases in child age increased odds of malaria whereas child sex had no association with malaria, which also follow previous research^78–80^.

One potential exception to this is that we found that spraying dwellings for mosquitoes and using a bed net were not strongly associated with reduced childhood malaria. In contrast, previous research has shown that bed net usage and indoor residual spraying are extremely effective in reducing the burden of childhood malaria when administered concomitantly as opposed to in isolation^81^. This apparent inconsistency could be resolved by considering potential proxy effects of other variables included in our framework; for instance, improving maternal education and wealth can improve adherence of malarial interventions such as bed-net usage^70–74^. Alternatively, our modelling framework may be less well suited to detecting such complex interactions or the presence of strongly non-linear effects^81,82^. Hence, it would be premature to suggest our results run contrary to studies that show high adherence to well supported malarial interventions, such as spraying dwellings and bed-net use^39^.

### Limitations

Our analysis is a retrospective cross-sectional analysis that is limited to the countries that have been included in DHS surveys between 2010 and 2015 that had no missing data on the variables we deemed important and incorporated within our analysis. Hence, our complete case analysis leads to exclusion of other countries such as Rwanda, Democratic Republic of Congo, and Uganda and for datasets conducted post 2015 given that these datasets may not hold data on key variables. For example, malaria control in Democratic Republic of Congo does not include indoor residual spraying and hence this survey will have been excluded as we aim to test for agriculture-disease associations whilst controlling for as many confounders as possible. In this instance, we assume that the exposure and/or confounders used within our analysis are missing not at random and therefore a complete case analysis is a valid approach^83^.

Although we have a strong underlying conceptual framework and aimed to control for a large number of potential confounders due to socio-economic and environmental factors, our analysis is still correlational in its approach and should be interpreted as such, preventing us from firmly implying causal relationships^20,45,56,82^. In addition, we chose to exclude interactions and focus on main effects in this study, and we did not explore the use of squared terms for key environmental variables such as temperature or precipitation to retain model simplicity but at the potential expense of overlooking strongly non-linear associations^42,67,84^.

Two key confounders that were also not controlled for was the type of crop and the presence or abundance of livestock. Previous research suggests that mosquitoes readily feed on natural sources of plant sugars, hence crop type could be an important confounder as mosquitoes feeding on peri-domestic plant- and fruit-derived sugar sources can change malaria transmission dynamics^62,85^. Livestock were not incorporated as a variable considering our analysis focusses on specific agricultural land uses (e.g., rainfed). However, it is important to note that livestock have been shown to be a zoo-prophylaxis for malaria in certain locations and livestock are also important as sources of income and nutrition that improve the well-being of the populations who have access to them^45,86^. We also conducted our analysis using temperature and precipitation variables from the WorldClim v1 dataset to control for climate conditions of each survey month based on long-term monthly averages. This assumes that the 2010–2015 period does not depart from long-term (1950–2000) climate in a given region for any sample within the included DHS dataset^45^.

Changes in agricultural systems have profound economic and demographic effects affecting malaria which may be bidirectional, non-linear, and cyclical. For instance, displacement of subsistence agriculturalists can lead to urbanisation. Adoption of more efficient agricultural techniques could also increase household wealth. Yet, improvements in housing due to improvements in wealth are likely to further accompany improvements in agricultural techniques^14^. Although we aimed to incorporate some of these factors (e.g., wealth, maternal education, sanitation, water source) as covariates in our analyses, our framework is limited to providing conditional estimates (i.e., estimates of what would be the effect if wealth were not modified) and does not estimate the overall effect of changes in agricultural techniques. Here, dynamic modelling which considers non-linearity, bi-directionality and cyclical processes may be useful in assessing the impact of changes in agricultural techniques on malaria transmission in future^20^.

Another limitation is the spatial resolution within our analysis and its impact on selecting explanatory variables. Deforestation across sub-Saharan Africa is largely due to shifting agriculture by marginal or smallholder farmers who employ agricultural methods on small (e.g., 1–100 ha) plots of land^87^. Although the maximum flight distance of a female, human blood-fed *A. gambiae* mosquito is around 10 km, field studies have shown that flights beyond 1.5 km are rare^88^. As stated in the methods, the displacement of rural clusters up to 5 km and urban clusters up to 2 km by the DHS is conducted for confidentiality reasons. However, this displacement alongside the use of 10 km resolution data within this analysis may not adequately capture the association between agriculture, malaria ecology or epidemiology and potential confounders at finer spatial scales and could also artificially amplify the relationship between agriculture and malaria or could lead to spatial autocorrelation^89^. Hence, further research is required to understand spatial displacement and whether such agriculture-malaria associations remain constant when factoring in spatial measurement bias^90^.

## Conclusion

On the basis of the associations we uncover in this analysis, agricultural expansion has the potential to increase childhood malaria across sub-Saharan Africa, which may undermine efforts to achieve malaria eradication. Rainfed cropland is often regarded as a more extensive and therefore sustainable land use, yet here is associated with increased odds of childhood malaria in rural areas^91,92^. On the other hand, irrigating cropland, which often serves to intensify agriculture but which has barriers to implementation (e.g. agronomic, hydrologic and economic), is often regarded as more environmentally unsustainable^93^, and could confer a somewhat reduced odds or not be associated with childhood malaria in rural areas^94–96^.Environmental interventions such as shifting from rainfed to irrigated cropland in rural areas or creating mosaics of natural vegetation within intensive cropland systems may reduce the burden of malaria that is attributable to specific agricultural land uses. Decision makers now require further evidence on the causality of the agriculture-malaria relationship which can aid in optimal design and cost-effectiveness of land use policy options in rural and urban systems and how these measures will impact multiple aspects of sustainability including but not limited to water availability, biodiversity loss, malaria eradication, carbon emissions, soil health and economic productivity.

## Methods

### Data

We used the Demographic and Health Surveillance Data (DHS) and Malaria Indicator Surveys (MIS) to compile datasets for analysis. These are nationally-representative household surveys that provide data for a wide range of monitoring and impact evaluation indicators in the areas of population, health, socio-economics and nutrition^1,42,45,65,97^. We analysed data for 12 sub-Saharan countries (consisting of 12 mutually exclusive datasets) from the years 2010 to 2015 with all variables that were hypothesised (see below) to be important risk factors for the childhood malaria whilst controlling for potential confounders. More countries are included in these datasets, but we restricted our analyses to those for which covariate data were the most complete (see Table 1).

The final dataset included 24,034 cases from respondents of households within clusters within each country between 2010 and 2015. For analysis, we compiled 27 socio-economic, environmental, and agricultural land use variables based on their hypothesised or known links to malaria from previous studies. Socio-economic variables included age, sex, education, whether a bed-net was used, whether the dwelling was sprayed with insecticide, wealth index, urban/rural, water source, sanitation type and population density (all extracted from the DHS data except for population density, which came from the Gridded Population of the World, Version 4 (GPWv4) dataset)^98^. Environmental variables included forest cover, forest loss, temperature, precipitation and elevation, all extracted from the Global Forest Change and WorldClim datasets^99–101^. Agricultural land use variables included rainfed cropland, irrigated or post flooding cropland and crop-dominated or veg-dominated mosaics (see below), extracted from the European Space Agency (ESA) Climate Change Initiative Land Cover (CCI-LC) dataset^102^.

The DHS program does not report exact coordinates for the clusters included in the survey, but randomly displaces the cluster coordinates up to 2 km for urban clusters and up to 5 km for rural clusters, with a further 1% of rural clusters displaced up to 10 km. This is done to ensure privacy protections of survey participants^45,65^. Hence, to address the possible displacement of the exact locations, all environmental data were resampled to a resolution of 10 km to approximate the environmental conditions for each household in the year of sampling. A 10-km radius also corresponds to the maximum flight distance of a female, human blood-fed *A gambiae* mosquito, representing the maximum extent at which human and specific mosquito populations can be expected to interact^6^.

### Variables

#### Malaria outcome variable

The DHS and MIS survey explicitly test for the presence or absence of malaria in children under 5 years using blood smear tests (BSTs) or rapid diagnostic tests (RDTs)^103–105^.

Typically, RDTs detect the HRP2 protein encoded by pfhrp2 and pfhrp3 genes. However, recent research suggests that specific populations in the Horn of Africa experience more than 90% gene deletion. Hence, in these populations, sensitivity of the RDT is limited and therefore not useful for detecting *P. falciparum* malaria^106^. Given that the selection of countries in our dataset did not include countries that experience high gene deletion and gene deletion was lower in the timeframe of timeframe of sampling (2010–2015), we opted to include malaria presence or absence detected by RDTs. Research further suggests that the two outcome variables (BSTs and RDTs) are well correlated (r = 0.58)^42^. Hence, we constructed a binary outcome variable that is equal to 1 if the child had malaria either diagnosed by BSTs or RDTs and 0 otherwise.

#### Agriculture variables

Agriculture has consistently been considered a potential risk factor for malaria infection in multiple geographical contexts^6,16,20,66,107^. However, research that investigates specific agricultural land use types (e.g., rainfed vs irrigated or post-flooding vs mosaic systems) and malaria infection is limited. To test the associations between differing agricultural land uses, we extracted land-use classifications for each cluster in the survey year from the ESA CCI-LC dataset^102^. Within this analysis, we focused on three agricultural production methods (rainfed, irrigated or post-flooding, and mosaic systems) as potential risk factors of malaria and therefore exclude all other land-cover classes that were no agricultural land uses or potential confounders (e.g., shrubland, grasslands, lichens, and mosses).

Rainfed cropland is defined as agriculture that relies on rainfall for water. Irrigated or post-flooding cropland is defined as land that is customarily supplied with water by artificial means or through flood mechanisms for growing plants. Cropland-natural vegetation mosaics with varying levels of coverage in the CCI-LC dataset are further stratified into either crop-dominant mosaic (i.e., more than 50% of the mosaic is dominated by cropland) or veg-dominant mosaics (i.e., more than 50% of the mosaic is dominated by natural vegetation) (Bontemps et al., 2013).

#### Forest cover and forest loss

Forest loss and forest cover have similarly been considered important factors in malaria ecology across sub-Saharan Africa^15,18,42,43^. In addition, agriculture is considered the leading driver of forest loss in sub-Saharan Africa^87^. We extracted forest loss and forest cover data from the Global Forest Change Dataset for each cluster in the survey year to capture forest cover change for pre-production agriculture^99^. This dataset is a published high resolution spatially explicit global raster of twenty-first century forest cover change at a 30-m resolution from 2001 to 2017. We do not include forest gain due to concerns on the reliability of these data, following previous studies^108^.

#### Socio-economic variables

##### Age and sex

Our dataset specifies the age (years) and sex of each child, allowing us to control for their commonly reported effects on malaria^109^.

##### Education and wealth

Education and wealth have previously been found to be important variables in malaria epidemiology and ecology across sub-Saharan Africa^73^. Therefore, maternal education and wealth were included as potential confounders. Maternal education is defined as the level of education of the mother of each child and was classified into three categories: No education, Primary, and Secondary. Wealth within the DHS data is a composite measure of a household’s living standard and is considered to be a surrogate of a household’s economic status^45,55^. The index places households into categories representing wealth quintiles, where the higher the wealth quintile, the higher the economic status of the household.

##### Rural/urban

Within our dataset, children are classified as either living in rural or urban clusters. The rural–urban context has also previously been shown to be a major determinant of malaria where the risk of malaria infection was shown to decline from rural areas through peri-urban settlements to urban central areas^44^. In addition, most of the global population growth this century is predicted to occur in Africa with dramatic changes to population densities in both rural and urban landscapes expected. Given the importance of the rural–urban context, in addition to the primary analyses, we performed an additional subgroup analysis to determine how the agriculture-malaria relationship may differ across rural or urban landscapes (see ‘Statistical analysis’ section below for further details).

##### Water source and sanitation

Drinking water and poor sanitation have previously been found to be risk factors of malaria infection in sub-Saharan Africa^76^. DHS identifies the main source of drinking water used by the household, and the type of sanitary facility primarily used by each household. We grouped the type of water and sanitation used by the household into dichotomous measures reflecting improved or unimproved sanitation and water source based on existing peer-reviewed literature^45^.

##### Bed-net use and spraying of dwellings

Insecticide-treated mosquito nets (ITNs) and indoor residual spraying (IRS) are the two most frequent interventions used to combat malaria in Africa^81,110^. Such interventions may confound the agriculture-malaria relationship and therefore have been included as potential confounding variables. The DHS and MIS datasets provide discrete variables for whether a child slept in a bed-net for sleeping (0 = no, 1 = all children, 2 = some children, 3 = no net in household) and whether the dwelling has been sprayed against mosquitoes in the last 12 months (0 = no, 1 = yes). We further recode whether a child slept in a bed net to whether a bed net was used (0 = no, 1 = some children used a bed net, 2 = all children used a bed net) where we assume no net in the household was equivalent to no use of a bed net.

##### Population density

Population density is an important factor in malaria epidemiology and the process of urbanization and accompanying demographic change is associated with decreased risks of infection due to reduction of suitable breeding grounds for malaria vectors through reduction of vegetative cover, water surfaces and other natural surfaces with building structures and other paved surfaces as well as through pollution of available breeding sites^44,111^. To control for potential confounding, we extracted and included data for each cluster using 2010 as an average year for population density using the Gridded Population of the World, Version 4 (GPWv4))^98^.

#### Environmental variables

Mean temperature and precipitation have been shown to be significant predictors of malaria in sub-Saharan Africa^16,40,84,112,113^. The temperature and precipitation variables in our dataset are the long-term (1950–2000) mean temperature (degrees Celsius) and precipitation (millimetres) in the cluster during the survey month. Both variables were sourced from the WorldClim v1 dataset, which provides monthly mean precipitation from interpolated station data over the period 1950–2000^100^. Although we extract precipitation and temperature data in the month and year of the conducted survey, we do not explicitly control for any other seasonal factors (e.g., crop seasonality)^88^.

Elevation is an appealing environmental proxy for a variety of fundamental dynamic ecological factors (e.g. temperature, humidity, precipitation, air pressure, sunshine, wind velocity, altitudinal farming) critical for mosquito development^69,114^. For these reasons, elevation also relates to both where certain crops are grown (e.g., in highland vs lowland regions) as well as suitability for malaria transmission^101^. While we aimed to include some of the proximal factors proxied by elevation directly into the analyses, we retained elevation in analyses to control for these other elevation-related potential confounders. We extracted elevation measurements from the Amazon Web Services Terrain Tiles dataset using the ‘elevatr’ package within R.

### Statistical analysis

#### Descriptive analysis

Our initial dataset consisted of approximately 2.3 million respondents based on all DHS and MIS surveys extracted in 2018. We excluded surveys that had no geo-referenced presence/absence of malaria tested through BSTs or RDTs alongside socio-economic variables. Variables (e.g., presence of specific malaria species, presence of soap/detergent, main housing material used, and type of bed net used) that were correlated with existing socio-economic variables such as sanitation, water sources, bed-net ownership were excluded. Variables describing livestock ownership type (e.g., cows, chickens, goats etc.) were also excluded due to a high level of missingness (> 95%) which was not random. Finally, we removed duplicate records and performed a complete case analysis that only included participants for which we had no missing data on the variables of interest. Baseline characteristics and descriptive characteristics were computed and are presented in the results^115^.

#### Multicollinearity

Multicollinearity arises in statistical models when two or more covariates are not statistically independent (i.e., correlated), leading to unstable estimates of variances of regression coefficients. To control for multi-collinearity we calculated the variance inflation factor (VIF), which represents the amount of variability of a covariate which is explained by other covariates^116^. We calculated the VIF for the candidate set of environmental, agricultural, and socio-economic variables. A methodological rule-of-thumb suggests that variables that have a VIF greater than 10 should be excluded^117^. No variables met this threshold for exclusion and VIF scores are presented alongside the results.

#### Hierarchical modelling

Data were analysed within a multi-model inference framework^45,65,118^, chosen to reduce the risk of overfitting, avoid the arbitrary stepwise approach to model selection and allow for the simultaneous assessment of different models and their associated hypotheses^119^. Within this framework, we used hierarchical models to control for covariates measured at different levels within the hierarchical dataset, thereby allowing the correction of biases in parameter estimates due to clustering of observations^45,120^.

The dataset is structured as individuals (level 1) that reside in households (level 2) that are in clusters (level 3) located within countries (level 4). Hence, we fitted a four-level hierarchical model using a binomial distribution for malaria presence/absence with a logit link function. We assume that there may be random variability across households, clusters and countries and therefore added a random effect at each of the four levels^45,121^.

We specified 81 candidate models using a *priori* hypotheses. These models included various combinations of socio-economic, agricultural land use, forest cover, forest loss, and climate explanatory variables. To specify an example candidate model (with all covariates), we observe M_ijkl_, a binary variable for malaria presence or absence for child i in household j in cluster k in country l. We define the probability of malaria equal to 1 as P_ijkl_ = Pr(M_ijkl_ = 1) and let P_ijkl_ be modelled using a logit link function. The four-level global model can be written as:

$${\text{Log}}[{\text{P}}_{{i{\text{jkl}}}} /(1 - {\text{P}}_{{{\text{ijkl}}}} )] = {\text{ }}\upbeta _{0} + {\text{ }}\upbeta _{1} {\text{Age}}_{{{\text{ijkl}}}} + \upbeta _{2} {\text{Sex}}_{{{\text{ijkl}}}} + \upbeta _{3} {\text{Wealth}}_{{{\text{jkl}}}} + \upbeta _{4} {\text{Education}}_{{{\text{jkl}}}} + \upbeta _{5} {\text{RuralUrban}}_{{{\text{kl}}}} + \upbeta _{6} {\text{Year}}_{{{\text{kl}}}} {\text{ }} + \upbeta _{7} {\text{Improve}}\;{\text{Sanitation}}_{{{\text{jkl}}}} + \upbeta _{8} {\text{Improved}}\;{\text{Water}}\;{\text{Source}}_{{{\text{jkl}}}} + \upbeta _{9} {\text{Used}}\;{\text{a}}\;{\text{Bednet}}_{{{\text{jkl}}}} + \upbeta _{{10}} {\text{Dwelling}}\;{\text{Sprayed}}_{{{\text{jkl}}}} + \upbeta _{{11}} {\text{Population}}\;{\text{Density}}_{{{\text{kl }}}} + \upbeta _{{12}} {\text{Temperature}}_{{{\text{kl}}}} + \upbeta _{{13}} {\text{Precipitation}}_{{{\text{kl}}}} + \upbeta _{{14}} {\text{Elevation}}_{{{\text{kl}}}} + \upbeta _{{15}} {\text{Forest}}\;{\text{Loss}}_{{{\text{kl}}}} + \upbeta _{{16}} {\text{Forest}}\;{\text{Cover}}_{{{\text{kl}}}} + \upbeta _{{17}} {\text{Rainfed}}\;{\text{Cropland}}_{{{\text{kl}}}} + \upbeta _{{18}} {\text{Irrigated/Post - Flooding}}\;{\text{Croplandkl}} + \in _{{{\text{0jkl}}}} + \in _{{0{\text{kl}}}} + \in _{{{\text{0l}}}}$$

where $\in _{{{\text{0jkl}}}}$ = household-level random intercept, independent across households, within clusters, within countries, $\in _{{{\text{0kl}}}}$ = cluster-level random intercept, independent across clusters, within countries, $\in _{{{\text{0kl}}}}$ = country-level random intercept, independent across countries.

To assess predictive accuracy, model averaging was conducted based on Akaike’s Information Criterion (AIC), where predictions were combined using Akaike weights based on the inclusion of all candidate models with an AIC less than 5 compared to the best performing model (defined as the model with the lowest AIC value)^122–125^.

As with demographic and economic transitions, landscapes often also follow a sequence of different land-use regimes: from pre-settlement natural vegetation to frontier clearing, then to subsistence agriculture and small-scale farms, and finally to intensive agriculture, urban areas, and protected recreational lands^126^. Hence, initial results at the regional level are expressed in odds ratios across differing land use segments reflecting this successional transition process, starting with natural vegetation, through to the mosaic (crop-dominated or veg-dominated) systems and finally intensive agricultural land use systems (rainfed, irrigated or post-flooding).

To assess the sensitivity of each variable to the probability of childhood malaria, we conducted a marginal effects analysis using a global model (e.g., using all available variables). Marginal effects measure the instantaneous effect that a change in a particular explanatory variable has on the predicted probability of the outcome (here malaria occurrence), when the other covariates are kept fixed^127^, equivalent to a univariate sensitivity analysis. In nonlinear models the marginal effects differ from the estimated coefficient as these depend on the values of the other explanatory variables, and in our case, also depend on the estimated random effects of the hierarchical model^45^.

Here, we calculated marginal effects using a global model to estimate the impact on the probability of childhood malaria of increasing exposure to each of the agricultural, environmental, forest cover change and socioeconomic variables. The interpretation of marginal effects differs for discrete and continuous variables. For discrete variables, the marginal effect corresponds to changes in each of these variables from 0 to 1 (e.g., no to yes responses or unimproved to improved states).

To assess whether geographical heterogeneity may influence or explain the association between differing agricultural land uses and the odds of childhood malaria, we finally performed a subgroup analysis within the multi-inference modelling and within the marginal effects’ analysis. This was done by stratifying our regional data set into rural and urban subgroups and re-running both model averaging and marginal effects analysis. Supplementary Information Table S3 provides a description of the variables included within each of the 81 a priori models. All analyses were conducted in R version 4.1.2 with the “lme4” package^128,129^. Finally, all methods were performed in accordance with relevant guidelines and regulations.

## Supplementary Information

Supplementary Information.

## Data Availability

The code and environmental data that support the findings of this study are available from the corresponding author upon suitable request. The primary health data used in this analysis are available from http://dhsprogram.com/Data.
