# Supplementary material for: Exploring agricultural land-use and childhood malaria associations in sub-Saharan Africa
Source: Sci Rep. 2022 Mar 8;12:4124. doi: 10.1038/s41598-022-07837-6 (PMC8904834; doi:10.1038/s41598-022-07837-6)

**Supplementary Information**

Contents

1. Data flow diagram
2. List of DHS and MIS surveys included in initial data amalgamation
3. Table of included DHS datasets and year of survey
4. Sub-Saharan Africa regional analysis
5. Urban and Rural subgroup analysis
6. Model structures
7. Correlation Matrix

Figure S1 – Data Flow Diagram

**3,371,952** individual records amalgamated from 104 DHS and MIS surveys in October 2018

Removal of following variables that had more than 95% missingness and subsequent complete case analysis thereby removing 49,605 additional records.

Creation of binary malaria outcome variable and removal of 3,284,343 records that do not have a geolocated malaria presence or absence.

**87,609** individual records with geolocated malaria presence and absence data

**24,253** unique and individual records with complete information on socioeconomics, climate, agriculture and forest loss and gain

List of DHS and MIS surveys

| # | DHS MIS Survey | Sample Size | # | DHS MIS Survey | Sample Size |
| --- | --- | --- | --- | --- | --- |
| 1 | AL5 | 4089 | 53 | LB6 | 24932 |
| 2 | AM6 | 3828 | 54 | LS4 | 12192 |
| 3 | AM7 | 1066 | 55 | LS5 | 12980 |
| 4 | AO5 | 33116 | 56 | LS6 | 10068 |
| 5 | AO7 | 20001 | 57 | MA4 | 23965 |
| 6 | BD4 | 28772 | 58 | MB4 | 4550 |
| 7 | BD5 | 24010 | 59 | MD5 | 32508 |
| 8 | BD6 | 31709 | 60 | MD6 | 40902 |
| 9 | BF4 | 12165 | 61 | ML4 | 224974 |
| 10 | BF6 | 32827 | 62 | ML5 | 29896 |
| 11 | BJ4 | 9663 | 63 | ML6 | 32193 |
| 12 | BJ6 | 25935 | 64 | MM7 | 2138 |
| 13 | BO5 | 29831 | 65 | MW4 | 73019 |
| 14 | BU6 | 35113 | 66 | MW5 | 64440 |
| 15 | CD5 | 20556 | 67 | MW6 | 14679 |
| 16 | CD6 | 44521 | 68 | MW7 | 41228 |
| 17 | CI6 | 14604 | 69 | MZ6 | 29313 |
| 18 | CM4 | 18529 | 70 | NG4 | 14094 |
| 19 | CM6 | 27097 | 71 | NG5 | 49393 |
| 20 | CO5 | 56056 | 72 | NG6 | 87196 |
| 21 | DR5 | 33233 | 73 | NM4 | 12218 |
| 22 | DR6 | 13235 | 74 | NM5 | 14218 |
| 23 | EG4 | 129765 | 75 | NM6 | 13330 |
| 24 | EG5 | 43969 | 76 | PE4 | 42755 |
| 25 | EG6 | 129344 | 77 | PE5 | 82210 |
| 26 | ET4 | 66548 | 78 | PE6 | 31311 |
| 27 | ET6 | 34719 | 79 | PH4 | 28897 |
| 28 | GA6 | 16203 | 80 | PH5 | 28770 |
| 29 | GH4 | 7245 | 81 | PK5 | 47642 |
| 30 | GH5 | 5958 | 82 | RW4 | 23725 |
| 31 | GH6 | 13667 | 83 | RW5 | 15586 |
| 32 | GN4 | 8539 | 84 | RW6 | 44215 |
| 33 | GN6 | 8486 | 85 | SL5 | 7387 |
| 34 | GU6 | 37283 | 86 | SL6 | 19028 |
| 35 | GY5 | 6776 | 87 | SN4 | 22954 |
| 36 | HN6 | 35930 | 88 | SN5 | 31490 |
| 37 | HT4 | 24268 | 89 | SN6 | 65147 |
| 38 | HT5 | 19596 | 90 | SZ5 | 8378 |
| 39 | HT6 | 23465 | 91 | TD6 | 45927 |
| 40 | ID4 | 49073 | 92 | TG6 | 12812 |
| 41 | JO4 | 33571 | 93 | TJ6 | 15420 |
| 42 | JO5 | 58052 | 94 | TL5 | 32982 |
| 43 | JO6 | 55671 | 95 | TZ5 | 25291 |
| 44 | KE4 | 22352 | 96 | TZ7 | 24560 |
| 45 | KE5 | 23362 | 97 | UG4 | 23861 |
| 46 | KE6 | 98770 | 98 | UG5 | 39757 |
| 47 | KH4 | 42961 | 99 | UG6 | 47294 |
| 48 | KH5 | 55463 | 100 | ZM5 | 17785 |
| 49 | KH6 | 19795 | 101 | ZM6 | 39024 |
| 50 | KM6 | 14405 | 102 | ZW5 | 12908 |
| 51 | KY6 | 14419 | 103 | ZW6 | 13600 |
| 52 | LB5 | 21499 | 104 | ZW7 | 15700 |

**Table of number of records by included DHS datasets and year of survey**

|  | 2010 | 2011 | 2012 | 2013 | 2014 | 2015 |
| --- | --- | --- | --- | --- | --- | --- |
| AO7 | 0 | 0 | 0 | 0 | 0 | 1361 |
| BF6 | 1263 | 0 | 0 | 0 | 0 | 0 |
| BJ6 | 0 | 149 | 1070 | 0 | 0 | 0 |
| BU6 | 0 | 0 | 2025 | 2 | 0 | 0 |
| CI6 | 0 | 128 | 923 | 0 | 0 | 0 |
| GH6 | 0 | 0 | 0 | 0 | 617 | 0 |
| GN6 | 0 | 0 | 561 | 0 | 0 | 0 |
| ML6 | 0 | 0 | 837 | 534 | 0 | 0 |
| MZ6 | 0 | 1903 | 0 | 0 | 0 | 0 |
| NG6 | 1290 | 0 | 0 | 0 | 0 | 2071 |
| SN6 | 449 | 604 | 592 | 1240 | 1669 | 1563 |
| TZ7 | 0 | 0 | 0 | 0 | 0 | 3314 |

Table S1 – Sub-Saharan Regional Analysis

|  | Standard Error | Odds Ratio | CI LOW | CI HIGH | DATATYPE |
| --- | --- | --- | --- | --- | --- |
| Year | 1.02141 | 0.95091 | 0.91224 | 0.99122 | Socioeconomics |
| Age | 1.01563 | 1.27654 | 1.23833 | 1.31594 | Socioeconomics |
| Population Density | 1.06942 | 0.68668 | 0.60204 | 0.78322 | Socioeconomics |
| Sex | 1.04569 | 0.99451 | 0.91113 | 1.08552 | Socioeconomics |
| Education | 1.26218 | 0.41418 | 0.26242 | 0.6537 | Socioeconomics |
| Used a bednet | 1.02875 | 1.00655 | 0.95215 | 1.06406 | Socioeconomics |
| Dwelling sprayed against mosquitoes | 1.11899 | 0.8684 | 0.69666 | 1.08249 | Socioeconomics |
| Wealth | 1.02647 | 0.67092 | 0.63742 | 0.70617 | Socioeconomics |
| Urban Rural | 1.08667 | 0.48121 | 0.40887 | 0.56636 | Socioeconomics |
| Water Source Unimproved | 1.0931 | 1.31613 | 1.10542 | 1.56701 | Socioeconomics |
| Sanitation Unimproved | 1.08081 | 1.18615 | 1.01856 | 1.38131 | Socioeconomics |
| Mean Temperature | 1.00202 | 1.00662 | 1.00264 | 1.01061 | Environmental |
| Precipitation | 1.00049 | 0.99921 | 0.99825 | 1.00017 | Environmental |
| Elevation | 1.08137 | 1.01881 | 0.87399 | 1.18763 | Environmental |
| Rainfed Cropland | 1.03806 | 1.12628 | 1.04677 | 1.21182 | Land Use |
| Irrigated or post-flooding cropland | 1.01714 | 1.02628 | 0.99266 | 1.06103 | Land Use |
| Crop-dominant Mosaic | 1.02133 | 0.95974 | 0.92085 | 1.00028 | Land Use |
| Veg-dominant Mosaic | 1.01821 | 1.02195 | 0.98644 | 1.05875 | Land Use |
| Forest Cover | 1.04427 | 1.34631 | 1.23672 | 1.46562 | Land Use |
| Forest Loss | 1.01332 | 0.98202 | 0.95688 | 1.00782 | Environmental |

Table s2 – Rural Urban Subgroup Analysis

|  | Rural | | | | Urban | | | |
| --- | --- | --- | --- | --- | --- | --- | --- | --- |
|  | Standard Error | Odds Ratio | CI LOW | CI HIGH | Standard Error | Odds Ratio | CI LOW | CI HIGH |
| Year | 1.02885 | 1.00434 | 0.94989 | 1.06191 | 1.07985 | 0.77637 | 0.66785 | 0.90253 |
| Age | 1.0181 | 1.28227 | 1.23797 | 1.32815 | 1.03063 | 1.26045 | 1.18808 | 1.33723 |
| Population Density | 1.29 | 0.2668 | 0.16197 | 0.43949 | 1.06571 | 0.82967 | 0.73238 | 0.93989 |
| Sex | 1.05326 | 1.01108 | 0.91331 | 1.11932 | 1.08927 | 0.91091 | 0.77035 | 1.07711 |
| Education | 1.30988 | 0.49615 | 0.29231 | 0.84215 | 1.59784 | 0.27861 | 0.11119 | 0.69811 |
| Has a bednet | 1.03374 | 0.99727 | 0.93448 | 1.06429 | 1.05133 | 1.00994 | 0.91555 | 1.11405 |
| Dwelling sprayed against mosquitoes | 1.14109 | 0.81454 | 0.62887 | 1.05502 | 1.23651 | 1.10767 | 0.73063 | 1.67927 |
| Wealth | 1.03044 | 0.76278 | 0.71924 | 0.80896 | 1.05472 | 0.51518 | 0.46409 | 0.57188 |
| Water Source Unimproved | 1.11736 | 1.39031 | 1.11854 | 1.7281 | 1.1815 | 1.04479 | 0.75346 | 1.44878 |
| Sanitation Unimproved | 1.09477 | 1.02082 | 0.85483 | 1.21905 | 1.18323 | 1.63955 | 1.17899 | 2.28001 |
| Mean Temperature | 1.0024 | 1.00697 | 1.00225 | 1.0117 | 1.00048 | 1.00008 | 0.99914 | 1.00102 |
| Precipitation | 1.0006 | 0.99815 | 0.99698 | 0.99933 | 1.0001 | 1.00012 | 0.99993 | 1.00032 |
| Elevation | 1.10141 | 0.8802 | 0.72839 | 1.06365 | 1.01729 | 0.99069 | 0.95795 | 1.02455 |
| Rainfed Cropland | 1.03731 | 1.1021 | 1.02575 | 1.18414 | 1.01107 | 0.99329 | 0.97209 | 1.01495 |
| Irrigated or post-flooding cropland | 1.02041 | 1.00763 | 0.96851 | 1.04834 | 1.04293 | 1.08766 | 1.00164 | 1.18107 |
| Crop-dominant Mosaic | 1.03462 | 0.91143 | 0.85262 | 0.97429 | 1.00796 | 1.00427 | 0.98879 | 1.01999 |
| Veg-dominant Mosaic | 1.02273 | 1.03944 | 0.99464 | 1.08625 | 1.00734 | 0.99617 | 0.98198 | 1.01056 |
| Forest Cover | 1.05524 | 1.22627 | 1.10362 | 1.36255 | 1.07661 | 1.62188 | 1.40339 | 1.87437 |
| Forest Loss | 1.01406 | 0.98301 | 0.95647 | 1.01029 | 1.02482 | 0.96789 | 0.92248 | 1.01554 |

Table S3 - List of model values

| Model | AIC | BIC | Log Likelihood | Deviance | Residuals | Equation |
| --- | --- | --- | --- | --- | --- | --- |
| africa0 | 17016 | 17162 | -8490 | 16980 | 24016 | malaria ~ year + age + pop_dense + sex + education + usedbednet + dwelling_sprayed_against_mosquitoes + wealth_index + URBAN_RURA + water_source + sanitation + mean_temp + prec + elevation + (1 \| country_code/cluster/household.) |
| africa1 | 17012 | 17190 | -8484 | 16968 | 24012 | malaria ~ year + age + pop_dense + sex + education + usedbednet + dwelling_sprayed_against_mosquitoes + wealth_index + URBAN_RURA + water_source + sanitation + mean_temp + prec + elevation + X10_baseline + X20_baseline + X30_baseline + X40_baseline + (1 \| country_code/cluster/household.) |
| africa2 | 17011 | 17164 | -8486 | 16973 | 24015 | malaria ~ year + age + pop_dense + sex + education + usedbednet + dwelling_sprayed_against_mosquitoes + wealth_index + URBAN_RURA + water_source + sanitation + mean_temp + prec + elevation + X10_baseline + (1 \| country_code/cluster/household.) |
| africa3 | 17018 | 17172 | -8490 | 16980 | 24015 | malaria ~ year + age + pop_dense + sex + education + usedbednet + dwelling_sprayed_against_mosquitoes + wealth_index + URBAN_RURA + water_source + sanitation + mean_temp + prec + elevation + X20_baseline + (1 \| country_code/cluster/household.) |
| africa4 | 17014 | 17168 | -8488 | 16976 | 24015 | malaria ~ year + age + pop_dense + sex + education + usedbednet + dwelling_sprayed_against_mosquitoes + wealth_index + URBAN_RURA + water_source + sanitation + mean_temp + prec + elevation + X30_baseline + (1 \| country_code/cluster/household.) |
| africa5 | 17018 | 17172 | -8490 | 16980 | 24015 | malaria ~ year + age + pop_dense + sex + education + usedbednet + dwelling_sprayed_against_mosquitoes + wealth_index + URBAN_RURA + water_source + sanitation + mean_temp + prec + elevation + X40_baseline + (1 \| country_code/cluster/household.) |
| africa6 | 16970 | 17131 | -8465 | 16930 | 24014 | malaria ~ year + age + pop_dense + sex + education + usedbednet + dwelling_sprayed_against_mosquitoes + wealth_index + URBAN_RURA + water_source + sanitation + mean_temp + prec + elevation + forest_cover + X10_baseline + (1 \| country_code/cluster/household.) |
| africa7 | 16977 | 17139 | -8469 | 16937 | 24014 | malaria ~ year + age + pop_dense + sex + education + usedbednet + dwelling_sprayed_against_mosquitoes + wealth_index + URBAN_RURA + water_source + sanitation + mean_temp + prec + elevation + forest_cover + X20_baseline + (1 \| country_code/cluster/household.) |
| africa8 | 16974 | 17136 | -8467 | 16934 | 24014 | malaria ~ year + age + pop_dense + sex + education + usedbednet + dwelling_sprayed_against_mosquitoes + wealth_index + URBAN_RURA + water_source + sanitation + mean_temp + prec + elevation + forest_cover + X30_baseline + (1 \| country_code/cluster/household.) |
| africa9 | 16979 | 17140 | -8469 | 16939 | 24014 | malaria ~ year + age + pop_dense + sex + education + usedbednet + dwelling_sprayed_against_mosquitoes + wealth_index + URBAN_RURA + water_source + sanitation + mean_temp + prec + elevation + forest_cover + X40_baseline + (1 \| country_code/cluster/household.) |
| africa10 | 16969 | 17155 | -8462 | 16923 | 24011 | malaria ~ year + age + pop_dense + sex + education + usedbednet + dwelling_sprayed_against_mosquitoes + wealth_index + URBAN_RURA + water_source + sanitation + mean_temp + prec + elevation + forest_cover + X10_baseline + X20_baseline + X30_baseline + X40_baseline + (1 \| country_code/cluster/household.) |
| africa11 | 16970 | 17140 | -8464 | 16928 | 24013 | malaria ~ year + age + pop_dense + sex + education + usedbednet + dwelling_sprayed_against_mosquitoes + wealth_index + URBAN_RURA + water_source + sanitation + mean_temp + prec + elevation + forest_cover + X10_baseline + forest_loss + (1 \| country_code/cluster/household.) |
| africa12 | 16978 | 17147 | -8468 | 16936 | 24013 | malaria ~ year + age + pop_dense + sex + education + usedbednet + dwelling_sprayed_against_mosquitoes + wealth_index + URBAN_RURA + water_source + sanitation + mean_temp + prec + elevation + forest_cover + X20_baseline + forest_loss + (1 \| country_code/cluster/household.) |
| africa13 | 16975 | 17145 | -8466 | 16933 | 24013 | malaria ~ year + age + pop_dense + sex + education + usedbednet + dwelling_sprayed_against_mosquitoes + wealth_index + URBAN_RURA + water_source + sanitation + mean_temp + prec + elevation + forest_cover + X30_baseline + forest_loss + (1 \| country_code/cluster/household.) |
| africa14 | 16979 | 17149 | -8469 | 16937 | 24013 | malaria ~ year + age + pop_dense + sex + education + usedbednet + dwelling_sprayed_against_mosquitoes + wealth_index + URBAN_RURA + water_source + sanitation + mean_temp + prec + elevation + forest_cover + X40_baseline + forest_loss + (1 \| country_code/cluster/household.) |
| africa15 | 16970 | 17164 | -8461 | 16922 | 24010 | malaria ~ year + age + pop_dense + sex + education + usedbednet + dwelling_sprayed_against_mosquitoes + wealth_index + URBAN_RURA + water_source + sanitation + mean_temp + prec + elevation + forest_cover + X10_baseline + X20_baseline + X30_baseline + X40_baseline + forest_loss + (1 \| country_code/cluster/household.) |
| africa16 | 17012 | 17174 | -8486 | 16972 | 24014 | malaria ~ year + age + pop_dense + sex + education + usedbednet + dwelling_sprayed_against_mosquitoes + wealth_index + URBAN_RURA + water_source + sanitation + mean_temp + prec + elevation + X10_baseline + forest_loss + (1 \| country_code/cluster/household.) |
| africa17 | 17019 | 17181 | -8490 | 16979 | 24014 | malaria ~ year + age + pop_dense + sex + education + usedbednet + dwelling_sprayed_against_mosquitoes + wealth_index + URBAN_RURA + water_source + sanitation + mean_temp + prec + elevation + X20_baseline + forest_loss + (1 \| country_code/cluster/household.) |
| africa18 | 17016 | 17177 | -8488 | 16976 | 24014 | malaria ~ year + age + pop_dense + sex + education + usedbednet + dwelling_sprayed_against_mosquitoes + wealth_index + URBAN_RURA + water_source + sanitation + mean_temp + prec + elevation + X30_baseline + forest_loss + (1 \| country_code/cluster/household.) |
| africa19 | 17021 | 17182 | -8490 | 16981 | 24014 | malaria ~ year + age + pop_dense + sex + education + usedbednet + dwelling_sprayed_against_mosquitoes + wealth_index + URBAN_RURA + water_source + sanitation + mean_temp + prec + elevation + X40_baseline + forest_loss + (1 \| country_code/cluster/household.) |
| africa20 | 17013 | 17199 | -8484 | 16967 | 24011 | malaria ~ year + age + pop_dense + sex + education + usedbednet + dwelling_sprayed_against_mosquitoes + wealth_index + URBAN_RURA + water_source + sanitation + mean_temp + prec + elevation + X10_baseline + X20_baseline + X30_baseline + X40_baseline + forest_loss + (1 \| country_code/cluster/household.) |
| africa21 | 17027 | 17149 | -8499 | 16997 | 24019 | malaria ~ year + age + pop_dense + sex + education + usedbednet + dwelling_sprayed_against_mosquitoes + wealth_index + URBAN_RURA + water_source + sanitation + (1 \| country_code/cluster/household.) |
| africa22 | 17023 | 17153 | -8496 | 16991 | 24018 | malaria ~ year + age + pop_dense + sex + education + usedbednet + dwelling_sprayed_against_mosquitoes + wealth_index + URBAN_RURA + water_source + sanitation + X10_baseline + (1 \| country_code/cluster/household.) |
| africa23 | 17029 | 17159 | -8499 | 16997 | 24018 | malaria ~ year + age + pop_dense + sex + education + usedbednet + dwelling_sprayed_against_mosquitoes + wealth_index + URBAN_RURA + water_source + sanitation + X20_baseline + (1 \| country_code/cluster/household.) |
| africa24 | 17026 | 17155 | -8497 | 16994 | 24018 | malaria ~ year + age + pop_dense + sex + education + usedbednet + dwelling_sprayed_against_mosquitoes + wealth_index + URBAN_RURA + water_source + sanitation + X30_baseline + (1 \| country_code/cluster/household.) |
| africa25 | 17029 | 17159 | -8499 | 16997 | 24018 | malaria ~ year + age + pop_dense + sex + education + usedbednet + dwelling_sprayed_against_mosquitoes + wealth_index + URBAN_RURA + water_source + sanitation + X40_baseline + (1 \| country_code/cluster/household.) |
| africa26 | 17025 | 17178 | -8493 | 16987 | 24015 | malaria ~ year + age + pop_dense + sex + education + usedbednet + dwelling_sprayed_against_mosquitoes + wealth_index + URBAN_RURA + water_source + sanitation + X10_baseline + X20_baseline + X30_baseline + X40_baseline + (1 \| country_code/cluster/household.) |
| africa27 | 16980 | 17142 | -8470 | 16940 | 24014 | malaria ~ year + age + pop_dense + sex + education + usedbednet + dwelling_sprayed_against_mosquitoes + wealth_index + URBAN_RURA + water_source + sanitation + forest_cover + X10_baseline + X20_baseline + X30_baseline + X40_baseline + (1 \| country_code/cluster/household.) |
| africa28 | 16980 | 17117 | -8473 | 16946 | 24017 | malaria ~ year + age + pop_dense + sex + education + usedbednet + dwelling_sprayed_against_mosquitoes + wealth_index + URBAN_RURA + water_source + sanitation + forest_cover + X10_baseline + (1 \| country_code/cluster/household.) |
| africa29 | 16987 | 17124 | -8476 | 16953 | 24017 | malaria ~ year + age + pop_dense + sex + education + usedbednet + dwelling_sprayed_against_mosquitoes + wealth_index + URBAN_RURA + water_source + sanitation + forest_cover + X20_baseline + (1 \| country_code/cluster/household.) |
| africa30 | 16984 | 17121 | -8475 | 16950 | 24017 | malaria ~ year + age + pop_dense + sex + education + usedbednet + dwelling_sprayed_against_mosquitoes + wealth_index + URBAN_RURA + water_source + sanitation + forest_cover + X30_baseline + (1 \| country_code/cluster/household.) |
| africa31 | 16988 | 17125 | -8477 | 16954 | 24017 | malaria ~ year + age + pop_dense + sex + education + usedbednet + dwelling_sprayed_against_mosquitoes + wealth_index + URBAN_RURA + water_source + sanitation + forest_cover + X40_baseline + (1 \| country_code/cluster/household.) |
| africa32 | 16981 | 17151 | -8469 | 16939 | 24013 | malaria ~ year + age + pop_dense + sex + education + usedbednet + dwelling_sprayed_against_mosquitoes + wealth_index + URBAN_RURA + water_source + sanitation + forest_cover + X10_baseline + X20_baseline + X30_baseline + X40_baseline + forest_loss + (1 \| country_code/cluster/household.) |
| africa33 | 16980 | 17126 | -8472 | 16944 | 24016 | malaria ~ year + age + pop_dense + sex + education + usedbednet + dwelling_sprayed_against_mosquitoes + wealth_index + URBAN_RURA + water_source + sanitation + forest_cover + X10_baseline + forest_loss + (1 \| country_code/cluster/household.) |
| africa34 | 16988 | 17133 | -8476 | 16952 | 24016 | malaria ~ year + age + pop_dense + sex + education + usedbednet + dwelling_sprayed_against_mosquitoes + wealth_index + URBAN_RURA + water_source + sanitation + forest_cover + X20_baseline + forest_loss + (1 \| country_code/cluster/household.) |
| africa35 | 16985 | 17130 | -8474 | 16949 | 24016 | malaria ~ year + age + pop_dense + sex + education + usedbednet + dwelling_sprayed_against_mosquitoes + wealth_index + URBAN_RURA + water_source + sanitation + forest_cover + X30_baseline + forest_loss + (1 \| country_code/cluster/household.) |
| africa36 | 16989 | 17134 | -8476 | 16953 | 24016 | malaria ~ year + age + pop_dense + sex + education + usedbednet + dwelling_sprayed_against_mosquitoes + wealth_index + URBAN_RURA + water_source + sanitation + forest_cover + X40_baseline + forest_loss + (1 \| country_code/cluster/household.) |
| africa37 | 17028 | 17157 | -8498 | 16996 | 24018 | malaria ~ year + age + pop_dense + sex + education + usedbednet + dwelling_sprayed_against_mosquitoes + wealth_index + URBAN_RURA + water_source + sanitation + forest_loss + (1 \| country_code/cluster/household.) |
| africa38 | 16977 | 17139 | -8469 | 16937 | 24014 | malaria ~ year + age + pop_dense + sex + education + usedbednet + dwelling_sprayed_against_mosquitoes + wealth_index + URBAN_RURA + water_source + sanitation + forest_cover + forest_loss + mean_temp + prec + elevation + (1 \| country_code/cluster/household.) |
| africa39 | 17018 | 17172 | -8490 | 16980 | 24015 | malaria ~ year + age + pop_dense + sex + education + usedbednet + dwelling_sprayed_against_mosquitoes + wealth_index + URBAN_RURA + water_source + sanitation + forest_loss + mean_temp + prec + elevation + (1 \| country_code/cluster/household.) |
| africa40 | 17024 | 17161 | -8495 | 16990 | 24017 | malaria ~ year + age + pop_dense + sex + education + usedbednet + dwelling_sprayed_against_mosquitoes + wealth_index + URBAN_RURA + water_source + sanitation + +X10_baseline + forest_loss + (1 \| country_code/cluster/household.) |
| africa41 | 17030 | 17167 | -8498 | 16996 | 24017 | malaria ~ year + age + pop_dense + sex + education + usedbednet + dwelling_sprayed_against_mosquitoes + wealth_index + URBAN_RURA + water_source + sanitation + +X20_baseline + forest_loss + (1 \| country_code/cluster/household.) |
| africa42 | 17026 | 17164 | -8496 | 16992 | 24017 | malaria ~ year + age + pop_dense + sex + education + usedbednet + dwelling_sprayed_against_mosquitoes + wealth_index + URBAN_RURA + water_source + sanitation + +X30_baseline + forest_loss + (1 \| country_code/cluster/household.) |
| africa43 | 17030 | 17167 | -8498 | 16996 | 24017 | malaria ~ year + age + pop_dense + sex + education + usedbednet + dwelling_sprayed_against_mosquitoes + wealth_index + URBAN_RURA + water_source + sanitation + +X40_baseline + forest_loss + (1 \| country_code/cluster/household.) |
| africa44 | 17025 | 17187 | -8493 | 16985 | 24014 | malaria ~ year + age + pop_dense + sex + education + usedbednet + dwelling_sprayed_against_mosquitoes + wealth_index + URBAN_RURA + water_source + sanitation + +X10_baseline + X20_baseline + X30_baseline + X40_baseline + forest_loss + (1 \| country_code/cluster/household.) |
| africa45 | 16977 | 17130 | -8469 | 16939 | 24015 | malaria ~ year + age + pop_dense + sex + education + usedbednet + dwelling_sprayed_against_mosquitoes + wealth_index + URBAN_RURA + water_source + sanitation + forest_cover + mean_temp + prec + elevation + (1 \| country_code/cluster/household.) |
| africa46 | 18324 | 18381 | -9155 | 18310 | 24027 | malaria ~ mean_temp + prec + elevation + (1 \| country_code/cluster/household.) |
| africa47 | 18294 | 18359 | -9139 | 18278 | 24026 | malaria ~ X10_baseline + X20_baseline + X30_baseline + X40_baseline + (1 \| country_code/cluster/household.) |
| africa48 | 18300 | 18340 | -9145 | 18290 | 24029 | malaria ~ X10_baseline + (1 \| country_code/cluster/household.) |
| africa49 | 18367 | 18407 | -9178 | 18357 | 24029 | malaria ~ X20_baseline + (1 \| country_code/cluster/household.) |
| africa50 | 18365 | 18405 | -9177 | 18355 | 24029 | malaria ~ X30_baseline + (1 \| country_code/cluster/household.) |
| africa51 | 18366 | 18406 | -9178 | 18356 | 24029 | malaria ~ X40_baseline + (1 \| country_code/cluster/household.) |
| africa52 | 18230 | 18270 | -9110 | 18220 | 24029 | malaria ~ forest_cover + (1 \| country_code/cluster/household.) |
| africa53 | 18365 | 18405 | -9177 | 18355 | 24029 | malaria ~ forest_loss + (1 \| country_code/cluster/household.) |
| africa54 | 18164 | 18213 | -9076 | 18152 | 24028 | malaria ~ forest_cover + X10_baseline + (1 \| country_code/cluster/household.) |
| africa55 | 18231 | 18279 | -9109 | 18219 | 24028 | malaria ~ forest_cover + X20_baseline + (1 \| country_code/cluster/household.) |
| africa56 | 18163 | 18236 | -9073 | 18145 | 24025 | malaria ~ forest_cover + X10_baseline + X20_baseline + X30_baseline + X40_baseline + (1 \| country_code/cluster/household.) |
| africa57 | 18232 | 18280 | -9110 | 18220 | 24028 | malaria ~ forest_cover + X30_baseline + (1 \| country_code/cluster/household.) |
| africa58 | 18232 | 18280 | -9110 | 18220 | 24028 | malaria ~ forest_cover + X40_baseline + (1 \| country_code/cluster/household.) |
| africa59 | 18300 | 18349 | -9144 | 18288 | 24028 | malaria ~ forest_loss + X10_baseline + (1 \| country_code/cluster/household.) |
| africa60 | 18366 | 18415 | -9177 | 18354 | 24028 | malaria ~ forest_loss + X20_baseline + (1 \| country_code/cluster/household.) |
| africa61 | 18365 | 18413 | -9176 | 18353 | 24028 | malaria ~ forest_loss + X30_baseline + (1 \| country_code/cluster/household.) |
| africa62 | 18365 | 18414 | -9177 | 18353 | 24028 | malaria ~ forest_loss + X40_baseline + (1 \| country_code/cluster/household.) |
| africa63 | 18295 | 18368 | -9139 | 18277 | 24025 | malaria ~ forest_loss + X10_baseline + X20_baseline + X30_baseline + X40_baseline + (1 \| country_code/cluster/household.) |
| africa64 | 18224 | 18272 | -9106 | 18212 | 24028 | malaria ~ forest_loss + forest_cover + (1 \| country_code/cluster/household.) |
| africa65 | 18248 | 18337 | -9113 | 18226 | 24023 | malaria ~ mean_temp + prec + elevation + X10_baseline + X20_baseline + X30_baseline + X40_baseline + (1 \| country_code/cluster/household.) |
| africa66 | 18256 | 18321 | -9120 | 18240 | 24026 | malaria ~ mean_temp + prec + elevation + X10_baseline + (1 \| country_code/cluster/household.) |
| africa67 | 18324 | 18388 | -9154 | 18308 | 24026 | malaria ~ mean_temp + prec + elevation + X20_baseline + (1 \| country_code/cluster/household.) |
| africa68 | 18323 | 18388 | -9154 | 18307 | 24026 | malaria ~ mean_temp + prec + elevation + X30_baseline + (1 \| country_code/cluster/household.) |
| africa69 | 18326 | 18391 | -9155 | 18310 | 24026 | malaria ~ mean_temp + prec + elevation + X40_baseline + (1 \| country_code/cluster/household.) |
| africa70 | 18171 | 18236 | -9078 | 18155 | 24026 | malaria ~ mean_temp + prec + elevation + forest_cover + (1 \| country_code/cluster/household.) |
| africa71 | 18323 | 18388 | -9154 | 18307 | 24026 | malaria ~ mean_temp + prec + elevation + forest_loss + (1 \| country_code/cluster/household.) |
| africa72 | 18106 | 18179 | -9044 | 18088 | 24025 | malaria ~ mean_temp + prec + elevation + forest_cover + X10_baseline + (1 \| country_code/cluster/household.) |
| africa73 | 18167 | 18240 | -9074 | 18149 | 24025 | malaria ~ mean_temp + prec + elevation + forest_cover + X20_baseline + (1 \| country_code/cluster/household.) |
| africa74 | 18173 | 18246 | -9077 | 18155 | 24025 | malaria ~ mean_temp + prec + elevation + forest_cover + X30_baseline + (1 \| country_code/cluster/household.) |
| africa75 | 18173 | 18246 | -9078 | 18155 | 24025 | malaria ~ mean_temp + prec + elevation + forest_cover + X40_baseline + (1 \| country_code/cluster/household.) |
| africa76 | 18098 | 18195 | -9037 | 18074 | 24022 | malaria ~ mean_temp + prec + elevation + forest_cover + X10_baseline + X20_baseline + X30_baseline + X40_baseline + (1 \| country_code/cluster/household.) |
| africa77 | 18257 | 18330 | -9119 | 18239 | 24025 | malaria ~ mean_temp + prec + elevation + forest_loss + X10_baseline + (1 \| country_code/cluster/household.) |
| africa78 | 18323 | 18396 | -9152 | 18305 | 24025 | malaria ~ mean_temp + prec + elevation + forest_loss + X20_baseline + (1 \| country_code/cluster/household.) |
| africa79 | 18323 | 18395 | -9152 | 18305 | 24025 | malaria ~ mean_temp + prec + elevation + forest_loss + X30_baseline + (1 \| country_code/cluster/household.) |
| africa80 | 18325 | 18398 | -9154 | 18307 | 24025 | malaria ~ mean_temp + prec + elevation + forest_loss + X40_baseline + (1 \| country_code/cluster/household.) |
| africa81 | 18248 | 18345 | -9112 | 18224 | 24022 | malaria ~ mean_temp + prec + elevation + forest_loss + X10_baseline + X20_baseline + X30_baseline + X40_baseline + (1 \| country_code/cluster/household.) |

Correlation Matrix


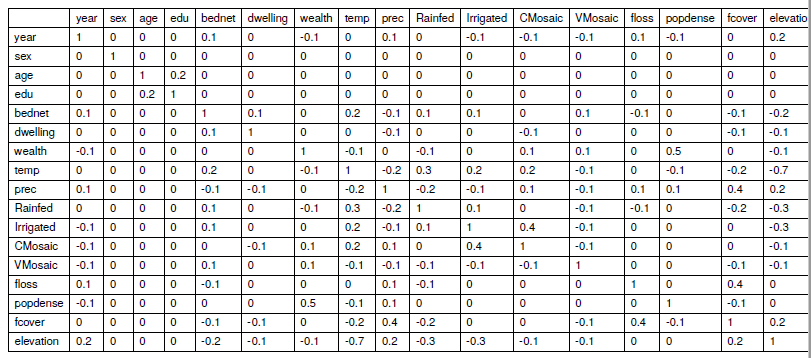

Supplement: Supplementary file 1 — Supplementary Information. [file 41598_2022_7837_MOESM1_ESM.docx]
